# Synthesis of Pelorol and Its Analogs and Their Inhibitory Effects on Phosphatidylinositol 3-Kinase

**DOI:** 10.3390/md14060118

**Published:** 2016-06-21

**Authors:** Yongjie Luo, Huixuan Chen, Jiang Weng, Gui Lu

**Affiliations:** 1 Institute of Medicinal Chemistry, School of Pharmaceutical Sciences, Sun Yat-sen University, Guangzhou 510006, China; 18666353586@163.com (Y.L.); chenhx33@mail2.sysu.edu.cn (H.C.); wengj2@mail.sysu.edu.cn (J.W.); 2 Institute of Human Virology, Sun Yat-sen University, Guangzhou 510080, China

**Keywords:** marine organisms, pelorol, total synthesis, PI3K inhibitor, anti-tumor activity

## Abstract

There are numerous biologically active substances with novel structures and unique physiological functions in marine organisms. These substances are important sources of new lead compounds. Pelorol is a natural product isolated from marine organisms that possesses a novel structure with high bioactivity. In this paper, the synthesis of pelorol has been completed, and the synthesis of some intermediates has been optimized and scaled up. Five pelorol analogs have also been prepared. Preliminary biological activity testing demonstrated that compounds **5** and **6** might be potential lead compounds for cancer therapy.

## 1. Introduction

**Figure 1:**
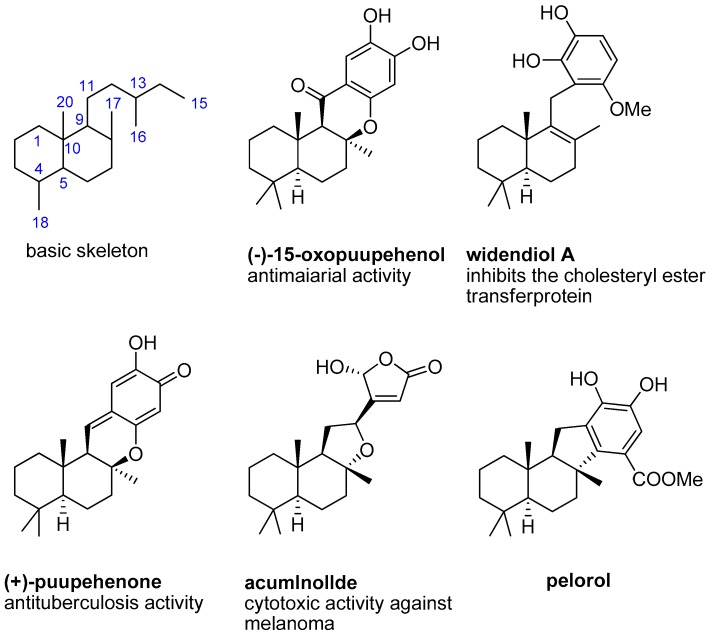
Representative bioactive compounds of labdane-type diterpenes.

Natural products play significant roles in drug discovery and development [1]. Marine organisms have been extensively investigated because they produce natural substances; for instance, 245 furanosesterterpenoids from marine organisms were described in at least 133 articles from 1996 to 2006 [2]. The number of publications may be higher if halogenated monoterpenes, sesquiterpenes, and polyterpenes extracted from the sponges are considered [3]. Labdane-type diterpenes are excellent examples of natural products with important pharmaceutical activities (Figure 1). Most of these derivatives possess significant biological properties such as anti-tumor, anti-HIV, anti-inflammatory, antifungal, and antibacterial activities [4,5,6,7,8].

Pelorol is a labdane-type diterpene isolated from the sponge [9,10]. It exhibits promising activity against trypanosome and *Plasmodium*. *In vitro* tests and mouse models reveals that pelorol is a src homology 2-containing inositol 5-phosphatase (SHIP) activators.

Phosphatidylinositol 3-kinases (PI3Ks) are a family of lipid kinases involved in cellular functions such as cell growth, proliferation, differentiation, motility, survival, and intracellular trafficking. The targeting of the PI3K pathway is one of the most promising approaches for the treatment of cancers. Moreover, SHIP can negatively regulate the PI3K pathway. The increased activity of SHIP can reduce the anti-apoptotic effect of the PI3K-Akt signaling pathway [11,12]. Thus, pelorol might also be a potential PI3K inhibitor.

However, natural pelorol is difficult to extract, and the synthetic of pelorol is rarely described. As far as we know, Andersen and co-workers [13] described a new strategy to synthesize pelorol with 5% overall yield in 2005. Their route is difficult to scale up because of numerous reaction steps and strict reaction conditions. In 2012, Baran and co-workers [14] developed another efficient method for the synthesis of pelorol, and they realized the gram-scale preparation of the key intermediate “borono-sclareolide”. Hence, new methods and technologies are still highly desirable for the synthesis, semi-synthesis, chemical modification and bioactivity assay of pelorol and related marine natural products, in order to provide technical support for the development of marine drug.

## 2. Results and Discussion

### 2.1. Chemistry

**Figure 2:**
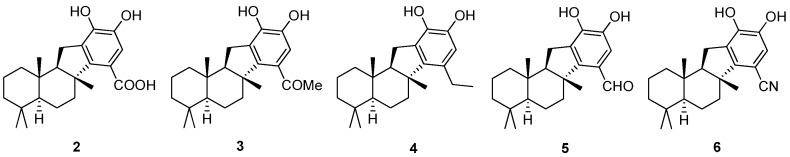
Pelerol analogs **2**–**6** in our work.

**Scheme 1:**
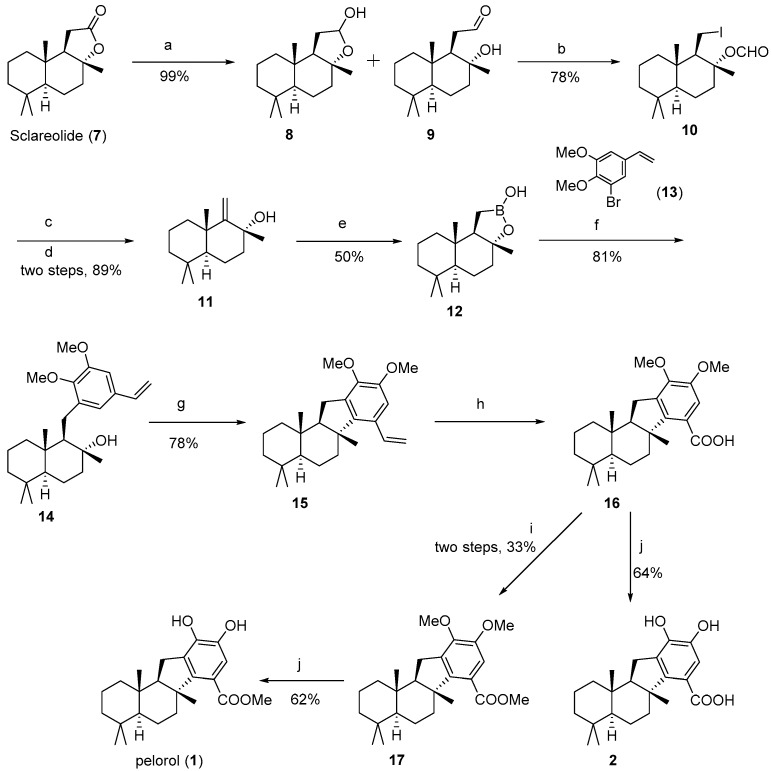
Synthesis of **1** and **2**. Reagents and conditions: (**a**) DIBAL, CH_2_Cl_2_, −78 °C, 1 h; (**b**) PIDA, I_2_, toluene, h*v*, 80 °C,1 h; (**c**) AgF, pyridine, room temperature (RT), 12 h; (**d**) K_2_CO_3_, MeOH, 0 °C–RT, 2 h; (**e**) BH_3_·THF, THF, 0 °C–RT, 12 h; (**f**) *S*-Phos, CsF, Pd(OAc)_2_, 1,4-dioxane, 50 °C, 18 h; (**g**) SnCl_4_, CH_2_Cl_2_, –78 °C, 1 h; (**h**) FeCl_3_·6H_2_O, TBHP, NaOH, CTAB, H_2_O, 80 °C, 48 h; (**i**) MeI, K_2_CO_3_, DMF, RT, 12 h; (**j**) BI_3_, CH_2_Cl_2_, –78 °C, 1 h.

In this paper, the synthesis of pelorol has been completed, optimized and scaled up based on Baran’s method (Scheme 1). Five pelorol analogs **2**–**6** have also been prepared (Figure 2).

Commercially available terpenoid (+)-sclareolide (**7**), which exhibits the same absolute configurations at C-5, C-9, and C-10 with pelorol (**1**), was used as starting material. **7** was reduced to a mixture of **8** and **9** (**8**/**9** = 4.59) with an excellent yield (99%); this mixture was used for the subsequent transformation without additional separation. Benzene is commonly used for iodination. Considering its toxicity, we replaced benzene with toluene and obtained product **10** with 78% yield. A sequential dehydroiodination/hydrolysis reaction (AgF in pyridine followed by K_2_CO_3_ in methanol) produced intermediate **11** with 89% overall yield. AgF is a precious metal salt and 1.5 equiv. of fresh AgF was essential in this reaction. To reduce the production costs, recycled AgF catalyst can be used, and in this case, 2.0 equiv. of catalyst was needed to ensure complete conversion. Hydroboration of **11** resulted in a mixture of diastereomers in 95% combined yield, and borono-sclareolide (**12**) can be separated by column chromatography in 50% yield.

At first, we followed Baran’s method and used aryl bromide with ethyl substituent (**18**) to couple with **12**, but the subsequent oxidation of intermediate **20** suffered from low yield. We have tried several different oxidants, such as DCC, PDC, ethylene oxide and potassium permanganate, but failed to improve the yield. Furthermore, subsequent reaction systems were quite complicated. Considering these problems, we changed the aryl bromide substrate to vinyl-substituted **13**, and found that the coupling reaction proceeded smoothly with up to 81% yield. What is more, this modification could remove two steps for the total synthesis of pelorol. The subsequent cyclization of **14** was performed at −78 °C to avoid the secondary reaction of the vinyl functional group. This cyclization provided the tetracyclic intermediate **15** in high yield (78%). Compounds **14** and **15** can also be prepared in gram-scale with comparable yields.

The oxidation of **15** via TBHP can generate **16** [15]. The synthesis of pelorol (**1**) was completed by the esterification of **16** with MeI and the selective cleavage of aryl methyl ether with BI_3_. The total yield of this route was 4.4%, which was similar to that of Andersen *et al*. [13]. The direct demethylation of **16** afforded compound **2** in 62% yield.

**Scheme 2:**
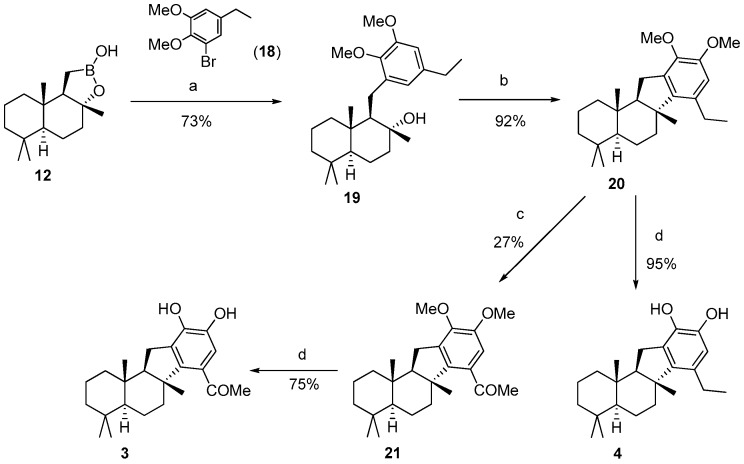
Synthesis of **3** and **4**. Reagents and conditions: (**a**) *S*-Phos, CsF, Pd(OAc)_2_, 1,4-dioxane, 50 °C, 12 h; (**b**) SnCl_4_, CH_2_Cl_2_, −20 °C, 1 h; (**c**) PCC, 40 °C, 20 h; (**d**) BI_3_, CH_2_Cl_2_, −78 °C, 1 h.

As we mentioned before, borono-sclareolide (**12**) could couple with ethyl-substituted **18** under Suzuki Coupling conditions developed by Buchwald [16]. A favorable yield of the coupling product **19** (73%) was obtained. The subsequent cyclization afforded **20** in excellent yield (92%). **20** was then oxidized by PCC to produce **21** in 27% yield; here the low yield might be due to the existence of two electron-donating methoxy groups on the phenyl ring of **20**. The demethylation of **20** and **21** provided **4** (95% yield) and **3** (75% yield), respectively (Scheme 2). The low yield in the latter reaction might be due to side reactions between ketone and BI_3_.

**Scheme 3:**
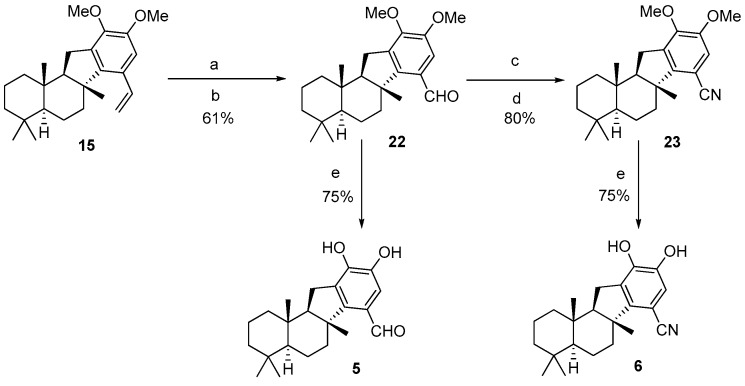
Synthesis of **5** and **6**. Reagents and conditions: (**a**) NMO, H_4_K_2_O_6_Os, THF:acetone:H_2_O=2:1:1, RT, 8 h; (**b**) NaIO_4_, CH_2_Cl_2_, 0 °C–RT, 3 h; (**c**) NH_2_OH·HCl, Et_3_N, RT, 4 h; (**d**) Cl_3_COCOOCCl_3_, CH_2_Cl_2_, RT, 8 h; (**e**) BI_3_, CH_2_Cl_2_, −78 °C, 1 h.

Intermediate **15** can be further oxidized to aryl aldehyde **22** under mild conditions [17,18]. Subsequent transformation of **22** via the formation of oxime and dehydration could yield **23** (80%) [19,20]. The demethylation of **22** and **23** afforded **5** (75% yield) and **6** (75% yield), respectively (Scheme 3). In this way, we have obtained five pelorol analogs **2**–**6**.

### 2.2. PI3K Enzymatic Activity Assay

All the newly prepared pelorol and analogs have been assessed *in vitro* in terms of biological activity against PI3Kα kinase, and the results are summarized in Table 1. Under the assay condition, IC_50_ value of pelorol was 38.17 μM. The carboxylic acid-substituted compound **2** showed slightly increased activity (20.70 μM). Among all these analogs, **3** (with acetyl substituent) and **5** (with aldehyde substituent) were the best PI3K inhibitors with IC_50_ of 8.76 μM and 5.06 μM, respectively [13]. Cyano-substituted compound **6** showed a significant decrease in activity (IC_50_ = 49.51 μM), while compound **4** with ethyl substituent on the phenyl ring exhibited poor performance (IC_50_ = 83.69 μM). We assumed that electronic effects might influence the inhibitory activity of compounds against PI3Kα. We have also found that dimethoxy-substituted compound **20** displayed weaker inhibitory activity when comparing with corresponding phenolic hydroxy-substituted compound **4**. The most efficient compound **5** was also subjected to PI3Kβ, PI3Kγ, PI3Kδ tests, and it exhibited significant inhibitory activity against PI3Kβ (IC_50_ = 8.90 μM).

### 2.3. Antiproliferative Assays in Vitro

Eight human cancer cell lines were used in the antiproliferative bioassay of pelorol and its analogs, the results were summarized in Table 2. The inhibitory activities of pelorol were poor. Compounds **2** and **4** also exhibited poor antiproliferative activities in these cancer cells, albeit **2** showed reasonable activity against PI3Kα. In contrast, compound **5** displayed the highest inhibitory activities and suppressed most of the cancer cells, especially MOLT-4 (leukemia cell), U937 (leukemia cell), and DU145 (prostate cancer cell), with IC_50_ at micromolar concentration levels. Although cyano-substituted compound **6** demonstrated moderate activity in kinase test, **6** could efficiently suppress a few tumor cells, especially MOLT-4 and DU145. Based on the above analysis, pelorol analogs **5** and **6** might be potential lead compounds for cancer therapy.

## 3. Materials and Methods

All commercial reagents and anhydrous solvents were obtained from commercial sources and were distilled from standard drying agents unless otherwise specified. ^1^H NMR spectra were recorded on a Bruker AVANCE III 400 spectrometer (Bruker Corp., Billerica, MA, USA) and referenced to tetramethylsilane in appropriate organic solutions. Chemical shifts were expressed as δ units using tetramethylsilane as internal standard (in NMR description: s, singlet; d, doublet; t, triplet; q, quartet; m, multiplet; bs, broad signal; *J*, joules; Hz, hertz). Mass spectra were measured with an Agilent 6120 spectrometer (Agilent Tech., Santa Clara, CA, USA) using an ESI+APCI source coupled to an Agilent 1200 HPLC system (Agilent Tech.) operating in reverse mode. High-resolution mass spectra were recorded on an ESI-ion trap mass spectrometer (Shimadzu LCMS-IT-TOF, Shimadzu, Kyoto, Japan), and the results agreed with the theoretical values to within 5 ppm. Purity of all biologically evaluated compounds was greater than 95%.

### 3.1. Chemistry

(*1R*,*2R*,*4aS*,*8aS*)-1-(Iodoethyl)-2,5,5,8a-tetramethyldecahydronaphthalen-2-yl formate (**10**) [14]: To a solution of (+)-sclareolide (10.00 g, 40.0 mmol, 1.0 equiv.) in 200 mL CH_2_Cl_2_ was added 1.0 M solution of DIBAL in heptane (48.0 mL, 48.0 mmol, 1.2 equiv.) dropwise at −78 °C. The reaction mixture was then stirred for another 60 min. Water was slowly added, the reaction mixture was warmed to room temperature and stirred overnight, extracted with CH_2_Cl_2_ (4 × 50 mL), washed with saturated brine (50 mL), dried over Na_2_SO_4_. Evaporation of the solvent afforded a white solid, which was used immediately in the next step.

The white solid in 90 mL anhydrous toluene was treated with PIDA (18.04 g, 56 mmol, 1.4 equiv.) and I_2_ (12.18 g, 48 mmol, 1.2 equiv.). The purple reaction mixture was vigorously stirred at 74 °C for 60 min under the irradiation of flood lamp (150 W). The reaction mixture was cooled, concentrated under reduced pressure, diluted with saturated brine, and extracted with EtOAc. The organic layers were combined, washed with saturated Na_2_S_2_O_3_, dried over Na_2_SO_4_. The solution was filtered and concentrated under reduced pressure. The residue was quickly diluted with anhydrous MeOH, stirred and cooled at −78 °C. The resulting white solid **10** (7.87 g, 78% yield) was collected via filtration. ^1^H NMR (400 MHz, CDCl_3_) δ: 8.02 (s, 1H), 3.36 (dd, *J* = 10.7, 2.9 Hz, 1H), 3.13 (dd, *J* = 10.7, 4.6 Hz, 1H), 2.55 (d, *J* = 12.7 Hz, 1H), 2.46 (d, *J* = 2.0 Hz, 1H), 1.93–1.85 (m, 2H), 1.69 (d, *J* = 3.5 Hz, 1H), 1.57 (s, 3H), 1.47 (d, *J* = 1.0 Hz, 3H), 1.38 (s, 1H), 1.25 (s, 2H), 1.07 (d, *J* = 2.3 Hz, 1H), 0.88 (s, 3H), 0.85 (d, *J* = 0.8 Hz, 3H), 0.79 (s, 3H). ^13^C NMR (101 MHz, CDCl_3_) δ: 160.3, 88.3, 62.1, 55.3, 41.6, 40.8, 39.6, 38.9, 33.2, 33.1, 21.4, 20.4, 19.7, 18.4, 14.8, −3.2. (Figure S1). 

(+)-Drim-9(11)-en-8-ol (**11**) [14]: A solution of compound **10** (2.32 g, 6.13 mmol, 1.0 equiv.) in anhydrous pyridine (20 mL) was treated with silver (I) fluoride (1.16 g, 9.2 mmol, 1.5 equiv.) at room temperature. The reaction flask was covered with aluminum foil to prevent light. The solution was stirred at room temperature for 12 h, then concentrated *in vacuo*, diluted with Et_2_O (20 mL), and filtered through celite pad. The dark grey solid (AgF) can be recycled. The filter cake was washed with Et_2_O (20 mL). The filtrate was combined, washed with saturated NaHCO_3_ and saturated Na_2_S_2_O_3_ ($V_{{\text{NaHCO}}_{3}}/V_{{\text{Na}}_{2}S_{2}O_{3}}=5:1$, 45 mL), then washed with brine (50 mL), dried over Na_2_SO_4_. The solution was filtered and concentrated under reduced pressure to afford unstable pale yellow syrup which was used immediately in the next step.

A solution of yellow syrup in 90 mL MeOH was treated with powdered, oven-dried K_2_CO_3_ (1.01 g, 7.35 mmol, 1.2 equiv.) at 0 °C. The reaction was warmed to room temperature and vigorously stirred for 2 h. The mixture was concentrated under reduced pressure, diluted with Et_2_O (20 mL), washed with H_2_O (60 mL), and extracted with Et_2_O (2 × 20 mL). The combined Et_2_O extracts were dried over Na_2_SO_4_, filtered and concentrated under reduced pressure to afford compound **11** as oyster white solid (1.18 g, 89% yield). ^1^H NMR (400 MHz, CDCl_3_) δ: 5.21 (s, 1H), 4.83 (s, 1H), 2.01 (d, *J* = 9.5 Hz, 1H), 1.79 (d, *J* = 12.6 Hz, 1H), 1.69 (t, *J* = 13.5 Hz, 2H), 1.53 (d, *J* = 14.8 Hz, 1H), 1.47 (s, 1H), 1.40 (s, 3H), 1.38–1.30 (m, 2H), 1.25 (s, 1H), 1.15 (d, *J* = 14.2 Hz, 1H), 1.08 (s, 3H), 0.98 (d, *J* = 11.0 Hz, 1H), 0.87 (s, 3H), 0.84 (s, 3H). ^13^C NMR (101 MHz, CDCl_3_) δ: 166.6, 103.7, 73.3, 53.5, 44.2, 41.8, 40.0, 39.0, 33.8, 33.2, 30.6, 29.68, 22.4, 21.6, 20.2, 19.1. (Figure S2).

Borono-sclareolide (**12**) [14]: Yield 50%, white solid. ^1^H NMR (400 MHz, CDCl_3_) δ: 6.62 (s, 1H), 1.95 (d, *J* = 11.6 Hz, 1H), 1.79–1.74 (m, 1H), 1.63 (d, *J* = 8.0 Hz, 2H), 1.56–1.50 (m, 1H), 1.42 (s, 3H), 1.38–1.34 (m, 1H), 1.33-1.23 (m, 1H), 1.17 (s, 4H), 0.98–0.90 (m, 2H), 0.85 (s, 3H), 0.82 (s, 3H), 0.80 (s, 3H), 0.75 (d, *J* = 7.6 Hz, 1H). ^13^C NMR (101 MHz, CDCl_3_) δ: 83.9, 61.6, 56.9, 42.5, 40.4, 39.5, 37.0, 33.3, 33.2, 23.1, 21.0, 20.9, 18.3, 14.6. (Figure S3).

(*1R*,*2R*,*4aS*,*8aS*)-1-(2,3-diMethoxy-5-vinylbenzyl)-2,5,5,8a-tetramethyldecahydronaphthalen-2-ol (**14**): To the borono-sclareolide **12** (1.00 g, 4.0 mmol, 1.0 equiv.) was added bromide **13** (1.94 g, 8 mmol, 2.0 equiv.), *S*-Phos (0.25 g, 0.6 mmol, 0.15 equiv.), CsF (1.82 g, 12 mmol, 3.0 equiv.) and 50 mL anhydrous dioxane. The solution was degassed for 20 min by bubbling N_2_ under sonication. Pd(OAc)_2_ (0.09 g, 0.4 mmol, 0.1 equiv.) was immediately added under N_2_, then the reaction mixture was heated at 50 °C for 18 h. The solution was cooled to room temperature and filtered through a celite pad, concentrated under reduced pressure. The crude product was purified by column chromatography on silica gel (petroleum ether/EtOAc = 30:1) to afford **14** (1.22 g, 80.5% yield) as colorless oil. ^1^H NMR (400 MHz, CDCl_3_) δ: 6.87 (d, *J* = 1.6 Hz, 1H), 6.81 (d, *J* = 1.7 Hz, 1H), 6.63 (dd, *J* = 17.5, 10.8 Hz, 1H), 5.63 (d, *J* = 17.5 Hz, 1H), 5.18 (d, *J* = 10.9 Hz, 1H), 3.86 (s, 3H), 3.85 (s, 3H), 2.84 (dd, *J* = 14.5, 5.1 Hz, 1H), 2.66 (s, 1H), 2.60 (d, *J* = 4.9 Hz, 1H), 1.86 (d, *J* = 12.5 Hz, 1H), 1.75 (d, *J* = 12.8 Hz, 1H), 1.62 (s, 3H), 1.41 (d, *J* = 14.0 Hz, 2H), 1.33 (d, *J* = 16.6 Hz, 2H), 1.28 (s, 3H), 1.10 (dd, *J* = 13.2, 3.6 Hz, 1H), 0.91 (s,3H), 0.85 (s, 3H), 0.83-0.81 (m, 1H), 0.80 (s, 3H). ^13^C NMR (101 MHz, CDCl_3_) δ: 152.6, 146.3, 138.1, 136.8, 133.5, 121.5, 112.9, 107.0, 73.7, 62.9, 60.7, 56.1, 55.7, 43.8, 41.8, 40.3, 39.5, 33.5, 33.3, 25.1, 24.2, 21.6, 20.3, 18.6, 15.5. (Figure S4).

(*4aS*,*6aR*,*11aR*,*11bS*)-9,10-diMethoxy-4,4,6a,11b-tetramethyl-7-vinyl-2,3,4,4a,5,6,6a,11,11a,11b-decahydro-1*H*-benzo[a]fluorene (**15**): To a stirred solution of **14** (232 mg, 0.6 mmol, 1.0 equiv.) in CH_2_Cl_2_ (60 mL), SnCl_4_ (0.6 mL) was added dropwise at −78 °C under N_2_. The resulting mixture was stirred for an additional 1 h. Upon the completion of the reaction (monitored by TLC), the solution was diluted with CH_2_Cl_2_ (90 mL) and poured into ice. The aqueous phase was extracted with CH_2_Cl_2_ (2 × 20 mL), the combined CH_2_Cl_2_ extracts were dried over Na_2_SO_4_, filtered and concentrated under reduced pressure. The residue was purified by column chromatography on silica gel (petroleum ether/EtOAc = 15:1) to give **15** as yellow-green crystal (173 mg, 78.3% yield). ^1^H NMR (400 MHz, CDCl_3_) δ: 7.06 (dd, *J* = 17.4, 10.9 Hz, 1H), 6.81 (s, 1H), 5.49 (dd, *J* = 17.3, 1.3 Hz, 1H), 5.17 (dd, *J* = 10.9, 1.3 Hz, 1H), 3.86 (s, 3H), 3.84 (s, 3H), 2.72 (dd, *J* = 14.7, 6.2 Hz, 1H), 2.56–2.47 (m, 1H), 2.42–2.35 (m, 1H), 1.73 (s, 3H), 1.61 (s, 2H), 1.43 (s, 2H), 1.26 (s, 1H), 1.18 (d, *J* = 4.3 Hz, 1H), 1.11 (s, 3H), 1.04 (s, 3H), 1.01–0.95 (m, 2H), 0.87 (s, 6H). ^13^C NMR (101 MHz, CDCl_3_) δ: 150.6, 145.5, 145.3, 135.8, 134.3, 128.3, 113.6, 107.7, 64.2, 60.4, 57.0, 56.0, 47.9, 42.5, 40.1, 39.5, 37.1, 33.4, 33.1, 25.3, 21.6, 21.1, 19.7, 18.4, 16.1. (Figure S5).

(*4aS*,*6aR*,*11aR*,*11bS*)-Methyl-9,10-dimethoxy-4,4,6a,11b-tetramethyl-2,3,4,4a,5,6,6a,11,11a,11b-decahydro-1*H*-benzo[a]fluorene-7-carboxylate (**17**): To a mixture of **15** (100 mg, 0.27 mmol, 1.0 equiv.) and ferric chloride hexahydrate (40 mg) was added TBHP (1.2 mL, 70% aqueous solution), CTAB (2 g) and 8 mL water. NaOH (40 mg, 1.0 mmol) was added after 3 h, and the mixture was heated at 90 °C for 48 h. After the completion of the reaction, dilute HCl and crushed ice were added. The mixture was extracted with EtOAc (2 × 50 mL), washed with saturated brine, dried over Na_2_SO_4_, then concentrated to dryness. The residue was dissolved in 2 mL DMF, then potassium carbonate (350 mg) and MeI (0.4 mL) were added. The solution was stirred overnight, quenched with 10 mL brine. The mixture was extracted with Et_2_O (2 × 20 mL), washed with brine, dried over MgSO_4_, and evaporated to dryness. The residue was purified by column chromatography on silica gel (petroleum ether/EtOAc = 8:1) to give **17** as colorless oil (34 mg, 33.3% yield). ^1^H NMR (400 MHz, CDCl_3_) δ: 7.03 (s, 1H), 3.88 (s, 3H), 3.86 (s, 3H), 3.84 (s, 3H), 2.71 (dd, *J* = 14.7, 6.2 Hz, 1H), 2.60–2.52 (m, 1H), 2.46 (dd, *J* = 9.1, 3.2 Hz, 1H), 1.66 (s, 2H), 1.40 (d, *J* = 6.2 Hz, 2H), 1.32 (s, 3H), 1.24 (s, 3H), 1.06 (s, 3H), 0.99 (d, *J* = 6.7 Hz, 1H), 0.94 (s, 1H), 0.90 (d, *J* = 2.8 Hz, 2H), 0.86 (s, 3H), 0.85 (s, 3H). ^13^C NMR (101 MHz, CDCl_3_) δ: 168.3, 149.8, 148.8, 148.3, 137.1, 121.2, 111.5, 64.8, 60.4, 57.1, 56.0, 51.8, 48.1, 42.5, 40.2, 37.1, 36.4, 33.4, 33.1, 25.3, 21.1, 20.0, 19.5, 18.35, 16.3. (Figure S6).

Pelorol (**1**): To a solution of methyl ester **17** (11 mg, 0.028 mmol, 1.0 equiv.) in 4 mL CH_2_Cl_2_ was added BI_3_ (129 mg, 0.336 mmol, 12 equiv.) in 2 mL CH_2_Cl_2_ dropwise at −78 °C under N_2_. The mixture was stirred for an additional 0.5 h, then poured into H_2_O and extracted with CH_2_Cl_2_ (2 × 50 mL). The combined CH_2_Cl_2_ extracts were dried over Na_2_SO_4_, filtered and concentrated under reduced pressure. The residue was purified by column chromatography on silica gel (petroleum ether/EtOAc = 3:1) to provide **1** as colorless oil (6.5 mg, 61.6% yield). ^1^H NMR (400 MHz, CDCl_3_) δ: 7.09 (s, 1H), 5.59 (s, 2H), 3.83 (s, 3H), 2.61 (d, *J* = 6.2 Hz, 1H), 2.54–2.47 (m, 2H), 1.66 (d, *J* = 6.2 Hz, 2H), 1.58–1.53 (m, 2H), 1.41 (dd, *J* = 8.8, 3.4 Hz, 2H), 1.25 (s, 3H), 1.23 (s, 3H), 1.05 (s, 3H), 0.98 (d, *J* = 4.5 Hz, 1H), 0.96–0.93 (m, 1H), 0.85 (d, *J* = 5.6 Hz, 6H). ^13^C NMR (101 MHz, CDCl_3_) δ: 168.3, 149.4, 143.7, 140.6, 130.0, 118.0, 114.8, 65.2, 57.1, 51.7, 48.5, 42.5, 40.2, 37.2, 36.5, 33.3, 33.1, 24.3, 21.1, 19.9, 19.5, 18.4, 16.3. (Figure S12). HRMS (ESI): *m/z* [M-H]^−^ calcd. for [C_23_H_32_O_4_]^−^: 371.2228, found: 371.2220. ${\left[a\right]}_{D}^{30}$ = −64.5 (*c* = 0.41, MeOH).

(*4aS*,*6aR*,*11aR*,*11bS*)-9,10-diHydroxy-4,4,6a,11b-tetramethyl-2,3,4,4a,5,6,6a,11,11a,11b-decahydro-1*H*-benzo[a]fluorene-7-carboxylic acid (**2**): To a solution of **16** (13 mg, 0.033 mmol, 1.0 equiv.) in 8 mL CH_2_Cl_2_ was slowly added BI_3_ (158 mg, 0.396 mmol, 12 equiv.) in 2 mL CH_2_Cl_2_ at −78 °C under N_2_. The mixture was stirred for an additional 0.5 h, then poured into H_2_O and extracted with CH_2_Cl_2_ (2 × 50 mL). The combined CH_2_Cl_2_ extracts were dried over Na_2_SO_4_, filtered and concentrated under reduced pressure. The residue was purified by column chromatography on silica gel (petroleum ether/EtOAc = 1:1) to provide **2** as colorless oil (7.7 mg, 64.1% yield). ^1^H NMR (400 MHz, DMSO-*d_6_*) δ: 12.15 (s, 1H), 8.98 (d, *J* = 113.4 Hz, 2H), 6.91 (s, 1H), 2.63 (d, *J* = 12.4 Hz, 1H), 2.34 (d, *J* = 13.2 Hz, 1H), 1.97 (s, 1H), 1.54 (s, 1H), 1.49–1.45 (m, 2H), 1.39–1.33 (m, 2H), 1.21 (s, 3H), 1.16 (d, *J* = 7.1 Hz, 2H), 1.11 (s, 3H), 0.99 (s, 3H), 0.90 (s, 1H), 0.81 (s, 6H). ^13^C NMR (101 MHz, DMSO-*d_6_*) δ: 169.1, 147.2, 145.2, 142.9, 130.3, 117.8, 115.1, 65.1, 60.2, 57.1, 48.1, 42.5, 37.1, 36.7, 33.6, 33.2, 24.9, 21.5, 20.1, 19.5, 18.4, 16.5. (Figure S13). HRMS (ESI): *m/z* [M-H]^−^ calcd. for [C_22_H_30_O_4_]^−^: 357.2071, found: 357.2059. ${\left[a\right]}_{D}^{30}$ = −11.61 (*c* = 0.37, MeOH).

(*1R*,*2R*,*4aS*,*8aS*)-1-(5-Ethyl-2,3-diMethoxybenzyl)-2,5,5,8a-tetramethyldecahydronaphthalen-2-ol (**19**) [14]: Yield 73%, colorless oil. ^1^H NMR (400 MHz, CDCl_3_) δ: 6.66 (s, 1H), 6.56 (s, 1H), 3.83 (s, 6H), 2.80 (s, 2H), 2.61–2.53 (m, 3H), 1.82 (s, 2H), 1.67 (s, 1H), 1.61 (s, 3H), 1.41 (d, *J* = 6.6 Hz, 2H), 1.33 (d, *J* = 15.8 Hz, 2H), 1.28 (s, 3H), 1.20 (t, *J* = 7.5 Hz, 3H), 1.09 (d, *J* = 3.4 Hz, 1H), 0.90 (s, 3H), 0.85 (s, 3H), 0.80 (s, 3H). ^13^C NMR (101 MHz, CDCl_3_) δ: 152.3, 144.3, 140.0, 137.6, 122.0, 109.6, 73.7, 62.8, 60.56, 56.1, 55.6, 43.7, 41.8, 40.3, 39.4, 33.5, 33.3, 28.8, 25.3, 24.3, 21.6, 20.3, 18.6, 15.7, 15.4. (Figure S7).

(*4aS*,*6aR*,*11aR*,*11bS*)-7-Ethyl-9,10-diMethoxy-4,4,6a,11b-tetramethyl-2,3,4,4a,5,6,6a,11,11a,11b-decahydro-1*H*-benzo[a]fluorene (**20**) [14]: Yield 92%, colorless oil. ^1^H NMR (400 MHz, CDCl_3_) δ: 6.53 (s, 1H), 3.85 (s, 3H), 3.84 (s, 3H), 2.76–2.68 (m, 2H), 2.59 (dd, *J* = 30.3, 10.4 Hz, 2H), 2.41 (d, *J* = 11.8 Hz, 1H), 1.81 (s, 1H), 1.79–1.73 (m, 3H), 1.61 (d, *J* = 13.1 Hz, 3H), 1.44 (d, *J* = 10.6 Hz, 3H), 1.29–1.25 (m, 3H), 1.13 (s, 3H), 1.07 (s, 3H), 1.00 (s, 1H), 0.90 (s, 6H). ^13^C NMR (101 MHz, CDCl_3_) δ: 150.4, 144.8, 143.6, 135.8, 133.7, 111.1, 64.3, 60.3, 57.1, 56.0, 47.9, 42.6, 40.2, 39.3, 37.1, 33.4, 33.1, 25.3, 24.7, 21.4, 21.2, 19.7, 18.4, 16.2, 16.1. (Figure S8).

1-((*4aS*,*6aR*,*11aR*,*11bS*)-9,10-diMethoxy-4,4,6a,11b-tetramet-hyl-2,3,4,4a,5,6,6a,11,11a,11b-decahydro-1*H*-benzo[a]fluoren-7-yl)ethanone (**21**) [14]: Yield 27%, white solid. ^1^H NMR (400 MHz, CDCl_3_) δ: 6.81 (s, 1H), 3.88 (s, 3H), 3.86 (s, 3H), 3.64 (d, *J* = 4.3 Hz, 1H), 2.71 (dd, *J* = 14.7, 6.2 Hz, 1H), 2.58 (d, *J* = 13.0 Hz, 1H), 2.52 (s, 3H), 2.32 (d, *J* = 12.5 Hz, 1H), 1.62 (d, *J* = 5.9 Hz, 6H), 1.40 (d, *J* = 9.1 Hz, 2H), 1.25 (s, 3H), 1.14 (s, 1H), 1.05 (s, 3H), 0.92 (s, 1H), 0.85 (d, *J* = 5.6 Hz, 6H). ^13^C NMR (101 MHz, CDCl_3_) δ: 201.9, 149.6, 147.9, 147.6, 137.4, 130.5, 110.0, 65.0, 60.5, 57.2, 56.2, 48.0, 42.5, 40.2, 37.1, 36.4, 33.4, 33.1, 30.3, 25.2, 21.1, 20.6, 19.4, 18.4, 16.3. (Figure S9).

1-((*4aS*,*6aR*,*11aR*,*11bS*)-9,10-diHydroxy-4,4,6a,11b-tetramethyl-2,3,4,4a,5,6,6a,11,11a,11b-decahydro-1*H*-benzo[a]fluoren-7-yl)ethanone (**3**): To a solution of **21** (34 mg, 0.088 mmol, 1.0 equiv.) in 5 mL CH_2_Cl_2_ was slowly added BI_3_ (413 mg, 1.05 mmol, 12 equiv.) in 2 mL CH_2_Cl_2_ at −78 °C under N_2_. The mixture was stirred for an additional 0.5 h, then poured into H_2_O and extracted with CH_2_Cl_2_ (2 × 30 mL). The combined CH_2_Cl_2_ extracts were dried over Na_2_SO_4_, filtered and concentrated. The residue was purified by column chromatography on silica gel (petroleum ether/EtOAc = 4:1) to provide **3** as colorless oil (23.5 mg, 75% yield). ^1^H NMR (400 MHz, CDCl_3_) δ: 6.94 (s, 1H), 5.80 (d, *J* = 157.3 Hz, 2H), 2.61 (dd, *J* = 14.2, 6.3 Hz, 1H), 2.51 (d, *J* = 12.9 Hz, 1H), 2.46 (s, 3H), 2.41 (d, *J* = 12.6 Hz, 1H), 1.64 (d, *J* = 5.9 Hz, 2H), 1.53 (dd, *J* = 9.7, 3.0 Hz, 2H), 1.64 (d, *J* = 5.9 Hz, 2H), 1.53 (dd, *J* = 9.7, 3.0 Hz, 2H), 1.42 (d, *J* = 4.8 Hz, 2H), 1.26 (s, 6H), 1.23 (s, 2H), 1.04 (s, 3H), 0.84 (d, *J* = 6.3 Hz, 6H). ^13^C NMR (101 MHz, CDCl_3_) δ: 201.5, 148.3, 143.3, 140.8, 130.3, 127.3, 113.7, 65.3, 57.2, 48.5, 42.5, 40.3, 37.1, 36.3, 33.4, 33.1, 29.7, 24.3, 21.1, 20.3, 19.5, 18.4, 16.3. (Figure S14). HRMS (ESI): *m/z* [M-H]^−^ calcd. for [C_23_H_32_O_3_]^−^: 355.2279, found: 355.2270. ${\left[a\right]}_{D}^{30}$ = −18.0 (*c* = 0.36, MeOH).

(*4aS*,*6aR*,*11aR*,*11bS*)-7-Ethyl-4,4,6a,11b-tetramethyl-2,3,4,4a,5,6,6a,11,11a,11b-decahydro-1*H*-benzo [a]fluorene-9,10-diol (**4**): To a solution of **20** (34 mg, 0.092 mmol, 1.0 equiv.) in 5 mL CH_2_Cl_2_ was slowly added BI_3_ (431 mg, 1.10 mmol, 12 equiv.) in 2 mL CH_2_Cl_2_ at −78 °C under N_2_. The mixture was stirred for an additional 0.5 h, then poured into H_2_O and extracted with CH_2_Cl_2_ (2 × 30 mL). The combined CH_2_Cl_2_ extracts were dried over Na_2_SO_4_, filtered and concentrated. The residue was purified by column chromatography on silica gel (petroleum ether/EtOAc = 4:1) to provide **4** as colorless oil (29.9 mg, 95% yield). ^1^H NMR (400 MHz, CDCl_3_) δ: 6.49 (s, 1H), 2.61 (s, 2H), 2.48 (s, 2H), 2.35 (s, 1H), 1.77 (s, 2H), 1.69 (s, 1H), 1.60–1.53 (m, 3H), 1.43 (d, *J* = 4.1 Hz, 2H), 1.25 (s, 3H), 1.18 (s, 3H), 1.08 (s, 3H), 1.03 (s, 3H), 0.87 (s, 6H). ^13^C NMR (101 MHz, CDCl_3_) δ: 144.8, 141.4, 137.9, 130.9, 128.7, 113.7, 64.6, 57.1, 48.1, 42.5, 40.1, 39.3, 37.1, 33.4, 33.1, 24.5, 24.2, 21.4, 21.1, 19.7, 18.4, 16.2, 15.9. (Figure S15). HRMS (ESI): *m/z* [M-H]^−^ calcd. for [C_23_H_34_O_2_]^−^: 341.2486, found: 341.2481. ${\left[a\right]}_{D}^{30}$ = −161.3 (*c* = 0.54, MeOH).

(*4aS*,*6aR*,*11aR*,*11bS*)-9,10-diMethoxy-4,4,6a,11b-tetramethyl-2,3,4,4a,5,6,6a,11,11a,11b-decahydro-1*H*-benzo[a]fluorene-7-carbaldehyde (**22**): To compound **15** (60 mg, 0.16 mmol, 1.0 equiv.) in 4 mL solvent (THF:acetone:H_2_O = 2:1:1) at room temperature was added NMO (0.7 mL) and H_4_K_2_O_6_Os (1 mg). The reaction mixture was stirred at room temperature for 12 h. Then the mixture was quenched with 5 mL saturated NaHS_2_O_3_, extracted with CH_2_Cl_2_ (2 × 30 mL). The combined CH_2_Cl_2_ extracts were dried over Na_2_SO_4_, filtered and concentrated. The residue was dissolved in 10 mL CH_2_Cl_2_, then NaIO_4_ (68.4 mg in 2 mL H_2_O) was added at room temperature for 3 h. The mixture was extracted with EtOAc (2 × 30 mL), washed with saturated brine (50 mL), dried over Na_2_SO_4_, filtered and concentrated. The residue was purified by column chromatography on silica gel (petroleum ether/EtOAc = 8:1) to provide **22** as colorless oil (36.8 mg, 61% yield). ^1^H NMR (400 MHz, CDCl_3_) δ: 10.32 (s, 1H), 7.21 (s, 1H), 3.88 (s, 3H), 3.82 (s, 3H), 2.70 (d, *J* = 6.2 Hz, 1H), 2.53 (s, 1H), 2.42 (d, *J* = 12.1 Hz, 1H), 1.87 (s, 1H), 1.73 (d, *J* = 12.8 Hz, 2H), 1.63 (d, *J* = 3.3 Hz, 1H), 1.56 (s, 1H), 1.52 (s, 1H), 1.46–1.40 (m, 1H), 1.37 (d, *J* = 13.3 Hz, 2H), 1.19 (s, 2H), 1.14 (s, 1H), 1.02 (s, 3H), 0.96 (s, 2H), 0.83 (s, 6H). ^13^C NMR (101 MHz, CDCl_3_) δ: 189.9, 153.2, 150.7, 136.1, 127.0, 108.1, 63.9, 60.4, 56.7, 55.9, 48.7, 42.4, 40.8, 40.1, 37.1, 33.3, 33.0, 25.5, 23.7, 21.1, 19.8, 18.3, 16.1. (Figure S10).

(*4aS*,*6aR*,*11aR*,*11bS*)-9,10-diMethoxy-4,4,6a,11b-tetramethyl-2,3,4,4a,5,6,6a,11,11a,11b-decahydro-1*H*-benzo[a]fluorene-7-carbonitrile (**23**): To a solution of **22** (49 mg, 0.13 mmol, 1.0 equiv.) and Et_3_N (25.6 mg, 0.14 mmol, 1.1 equiv.) in 2 mL CH_2_Cl_2_ was slowly added NH_2_OH·HCl (10 mg, 1.4 mmol, 10 equiv.) at 0 °C. The mixture was stirred for an additional 4 h at room temperature. Then Cl_3_COCOOCCl_3_ (43 mg, 0.15 mmol, 1.2 equiv.) was added, releasing a large number of bubbles. The reaction mixture was stirred at room temperature for 12 h, then extracted with CH_2_Cl_2_ (2 × 50 mL). The combined CH_2_Cl_2_ extracts were dried over Na_2_SO_4_, filtered and concentrated. The residue was purified by column chromatography on silica gel (petroleum ether/EtOAc = 8:1) to provide **23** as colorless oil (38.7 mg, 79.6% yield). ^1^H NMR (400 MHz, CDCl_3_) δ: 6.86 (s, 1H), 3.91 (s, 3H), 3.83 (s, 3H), 2.71 (d, *J* = 6.2 Hz, 1H), 2.62 (s, 1H), 2.53 (s, 1H), 1.82–1.76 (m, 1H), 1.72 (d, *J* = 6.5 Hz, 2H), 1.61 (s, 2H), 1.47 (s, 2H), 1.25 (d, *J* = 5.4 Hz, 2H), 1.14 (s, 3H), 1.04 (s, 3H), 0.99 (s, 1H), 0.95 (d, *J* = 10.3 Hz, 1H), 0.88 (s, 6H). ^13^C NMR (101 MHz, CDCl_3_) δ: 151.8, 149.5, 148.8, 135.5, 117.0, 112.9, 98.2, 63.1, 59.5, 56.2, 55.2, 46.3, 41.5, 39.1, 36.1, 35.8, 32.4, 32.1, 24.4, 20.8, 20.1, 18.3, 17.2, 15.1. (Figure S11).

(*4aS*,*6aR*,*11aR*,*11bS*)-9,10-diHydroxy-4,4,6a,11b-tetramethyl-2,3,4,4a,5,6,6a,11,11a,11b-decahydro-1*H*-benzo[a]fluorene-7-carbaldehyde (**5**): To a solution of **22** (34 mg, 0.092 mmol, 1.0 equiv.) in 5 mL CH_2_Cl_2_ was slowly added BI_3_ ( 431 mg, 1.10 mmol, 12 equiv.) in 2 mL CH_2_Cl_2_ at −78 °C under N_2_. The mixture was stirred for an additional 0.5 h, then poured into H_2_O and extracted with CH_2_Cl_2_ (2 × 30 mL). The combined CH_2_Cl_2_ extracts were dried over Na_2_SO_4_, filtered and concentrated. The residue was purified by column chromatography on silica gel (petroleum ether/EtOAc = 4:1) to provide **5** as colorless oil (23.5 mg, 75% yield). ^1^H NMR (400 MHz, CDCl_3_) δ: 10.26 (s, 1H), 7.37 (s, 1H), 6.80 (s, 1H), 6.14 (s, 1H), 2.72 (dd, *J* = 14.7, 5.9 Hz, 1H), 2.54 (d, *J* = 13.4 Hz, 2H), 1.92 (s, 1H), 1.76 (s, 3H), 1.58 (d, *J* = 12.1 Hz, 1H), 1.43 (d, *J* = 11.4 Hz, 2H), 1.26 (s, 2H), 1.22 (s, 3H), 1.07 (s, 3H), 1.00 (d, *J* = 13.8 Hz, 2H), 0.88 (s, 6H). ^13^C NMR (101 MHz, CDCl_3_) δ: 190.5, 154.5, 147.3, 142.0, 129.2, 124.3, 111.2, 64.2, 56.8, 49.0, 42.4, 40.7, 40.1, 37.1, 33.3, 33.1, 24.6, 23.8, 21.1, 19.8, 18.3, 16.1. (Figure S16). HRMS (ESI): *m/z* [M-H]^−^ calcd. for [C_22_H_30_O_3_]^−^: 341.2122, found: 341.2107. ${\left[a\right]}_{D}^{30}$ = 48.6 (*c* = 0.23, MeOH).

(*4aS*,*6aR*,*11aR*,*11bS*)-9,10-diHydroxy-4,4,6a,11b-tetramethyl-2,3,4,4a,5,6,6a,11,11a,11b-decahydro-1*H*-benzo[a]fluorene-7-carbonitrile (**6**): To a solution of **23** (11 mg, 0.03 mmol, 1.0 equiv.) in 5 mL CH_2_Cl_2_ was slowly added BI_3_ (140 mg, 0.36 mmol, 12 equiv.) in 1 mL CH_2_Cl_2_ at −78 °C under N_2_. The mixture was stirred for an additional 0.5 h, then poured into H_2_O. The solution was extracted with CH_2_Cl_2_ (2 × 30 mL). The combined CH_2_Cl_2_ extracts were dried over Na_2_SO_4_, filtered and concentrated. The residue was purified by column chromatography on silica gel (petroleum ether/EtOAc = 3:1) to provide **6** as colorless oil (7.6 mg, 75% yield). ^1^H NMR (400 MHz, CDCl_3_) δ: 6.89 (s, 1H), 4.13 (d, *J* = 7.2 Hz, 2H), 2.60 (dd, *J* = 20.5, 8.7 Hz, 2H), 2.45 (s, 1H), 2.06 (s, 1H), 1.73 (d, *J* = 12.9 Hz, 3H), 1.40 (s, 1H), 1.25 (s, 3H), 1.10 (s, 2H), 1.02 (s, 2H), 0.86 (s, 9H). ^13^C NMR (101 MHz, CDCl_3_) δ: 152.9, 145.6, 141.8, 129.8, 118.3, 116.7, 95.3, 64.4, 57.2, 47.6, 42.5, 40.1, 37.0, 36.8, 33.4, 33.1, 24.5, 21.9, 21.1, 19.4, 18.3, 16.2. (Figure S17). HRMS (ESI): *m/z* [M-H]^−^ calcd. for [C_22_H_29_NO_2_]^−^: 338.2126, found: 338.2119. ${\left[a\right]}_{D}^{30}$ = 10.2 (*c* = 0.53, MeOH).

### 3.2. Kinase Assays of PI3K

PI3K reactions were performed in 50 mM HEPES at pH 7.5 with 1 mM EGTA, 100 mM NaCl, 3 mM MgCl_2_, 2 mM DTT, and 0.03% CHAPS. All tested compounds were dissolved in 100% DMSO. PIP2 and ATP were used as substrates, and the final reaction volume was 10 μL. To evaluate the PI3Kα inhibitors, 1.65 nM enzyme, 50 μM PIP2, and 25 μM ATP were used for every 10 μL reaction volume with inhibitor concentrations ranging from 0.5 nM to 10 μM. After incubating for 1 h at room temperature, the reactions were quenched by adding 10 μL of Kinase-Glo reagent. Raw data were collected from Flexstation program.

### 3.3. Antiproliferative Assays in Vitro

The following cell lines, MCF-7, SK-BR-3, K562, Hela, MOLT-4, DU145, U937, and PC3 were purchased from Shanghai Cell Bank, Chinese Academy of Sciences. The cells were maintained at 37 °C in a 5% CO_2_ incubator in DMEM (Gibco, Invitrogen, Carlsbad, CA, USA) or RPMI1640 (Gibco, Invitrogen) containing 10% FBS. Cell proliferation was determined by CCK8 assay (DOjinDo, Kyoto, Japan). Cells were seeded at a density of 800–1000 cells/well in 384 well plates and treated with solvent control or various concentration of compounds. After 72 h incubation, CCK8 reagent was added and absorbance was measured at 450 nm by using Envision 2104 multi-label Reader (Perkin Elmer, Waltham, MA, USA). Dose-response curves were plotted to determine the IC_50_ values using Prism 5.0 (Graph Pad software Inc., San Diego, CA, USA).

## 4. Conclusions

Pelorol is natural product isolated from marine organisms, which possesses novel structure with high bioactivity. To solve the source problem of pelorol, we have developed an efficient and practical synthetic route for the preparation of pelorol in large scale. Moreover, the synthesis of some key intermediates has been optimized and scaled up. Five pelorol analogs have been prepared, their PI3K and tumor cell inhibitory activities have also been evaluated. This work is useful for further route optimization, structural modifications, activity assays and large-scale preparation of pelorol and related analogs.

## Figures and Tables

**Table 1:** Inhibition of pelorol and analogs against PI3Kα (μM) ^a^.

Compound	1	2	3	4	5 ^b^	6	20
IC_50_	38.17	20.70	8.76	83.69	5.06	49.51	>100

^a^ Average of three replicates; ^b^ For compound **5**, IC_50_ value for PI3Kβ is 8.90 μM, for PI3Kγ is >100 μM, for PI3Kδ is >100 μM.

**Table 2:** Relative IC_50_ values (μM) of pelorol and analogs against tumor cell lines ^a^.

Compound	1	2	3	4	5	6
MCF-7	>30	>30	>30	>30	38.9	16.5
K562	>30	>30	>30	>30	16.11	>30
Hela	>30	N.D. ^b^	>30	>30	13.15	12.05
DU145	72.69	>30	>30	>30	9.01	8.13
MOLT-4	>30	N.D.	22.3	>30	4.27	2.89
SK-BR-3	>30	N.D.	>30	>30	19.14	>30
U937	>30	N.D.	>30	>30	5.99	12.85
PC3	>30	>30	>30	>30	18.36	>30

^a^ Average of three replicates; ^b^ N.D.: not determined.

## Supplementary Materials

The following are available online at www.mdpi.com/link, Figures S1–S17: ^1^H and ^13^C NMR spectra of compounds.

## Abbreviations

The following abbreviations are used in this manuscript:

CHAPS	3-[(3-Cholamidopropyl)dimethylammonio]-1-propane sulfonate
CTAB	Hexadecyl trimethyl ammonium bromide
DCC	Dicyclimide
DIBAL	Diisobutylaluminium hydride
DMEM	Dulbecco minimum essential medium
DTT	Dithiothreitol in Ethyl acetate
EGTA	Ethylenebis(oxyethylenenitrilo)tetraacetic acid
FBS	Fetal calf serum
HEPES	2-[4-(2-Hydroxyethyl)-1-piperazinyl]ethanesulfonic acid
NMO	4-Methylmorpholine *N*-oxide
PCC	Pyridinum chlorochromate
PDC	Pyridinium dichromate
PIDA	Iodobenzene diacetate
TBHP	*tert*-Butylhydroperoxide
TLC	Thin layer chromatography
S-Phos	2-Dicyclohexylphosphino-2′,6′-diMethoxy-1,1′-biphenyl
