# Supplementary material for: Synthesis of Pelorol and Its Analogs and Their Inhibitory Effects on Phosphatidylinositol 3-Kinase
Source: Mar Drugs. 2016 Jun 21;14(6):118. doi: 10.3390/md14060118 (PMC4926077; doi:10.3390/md14060118)
Supplement: Supplementary file 1 [file marinedrugs-14-00118-s001.pdf]

Supplementary Materials: Synthesis of Pelorol and Its Analogs and Their Inhibitory Effects on Phosphatidylinositol 3-Kinase

Yongjie Luo, Huixuan Chen, Jiang Weng and Gui Lu

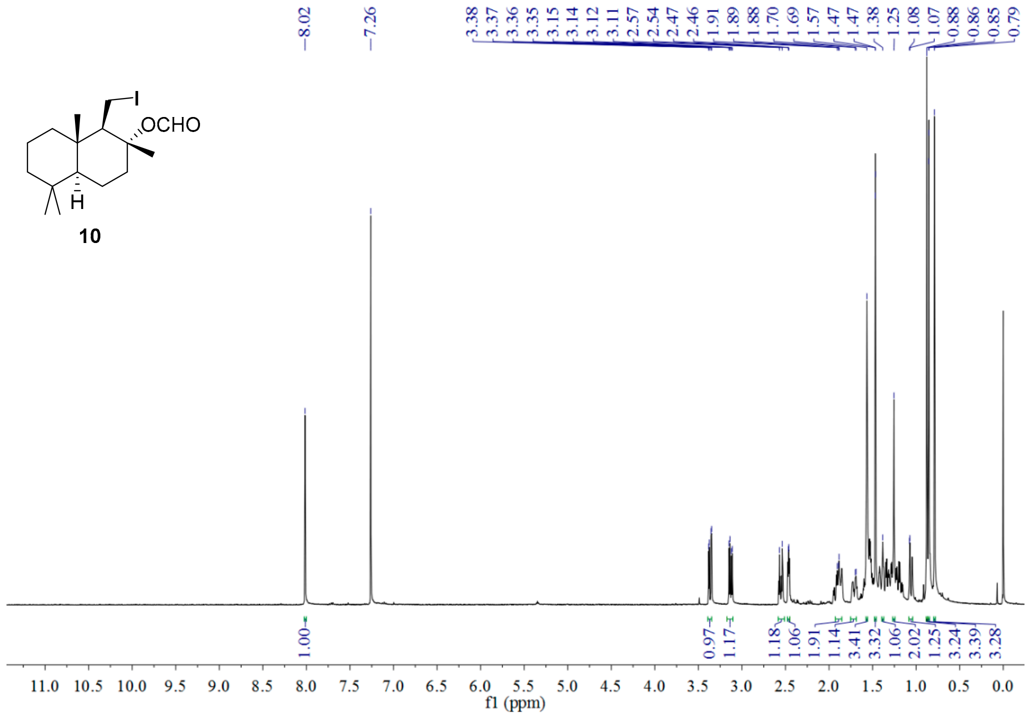

(**a**)

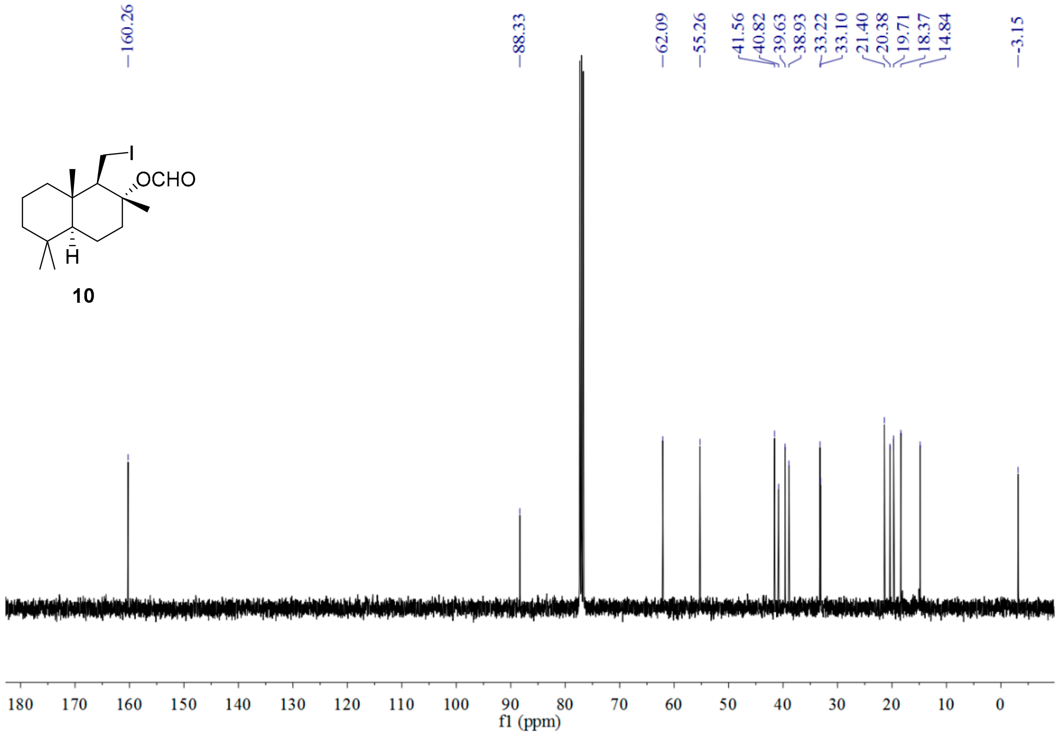

(**b**)

Figure S1. (**a**) ^{1}H NMR spectrum of compound **10**; (**b**) ^{13}C NMR spectrum of compound **10**.

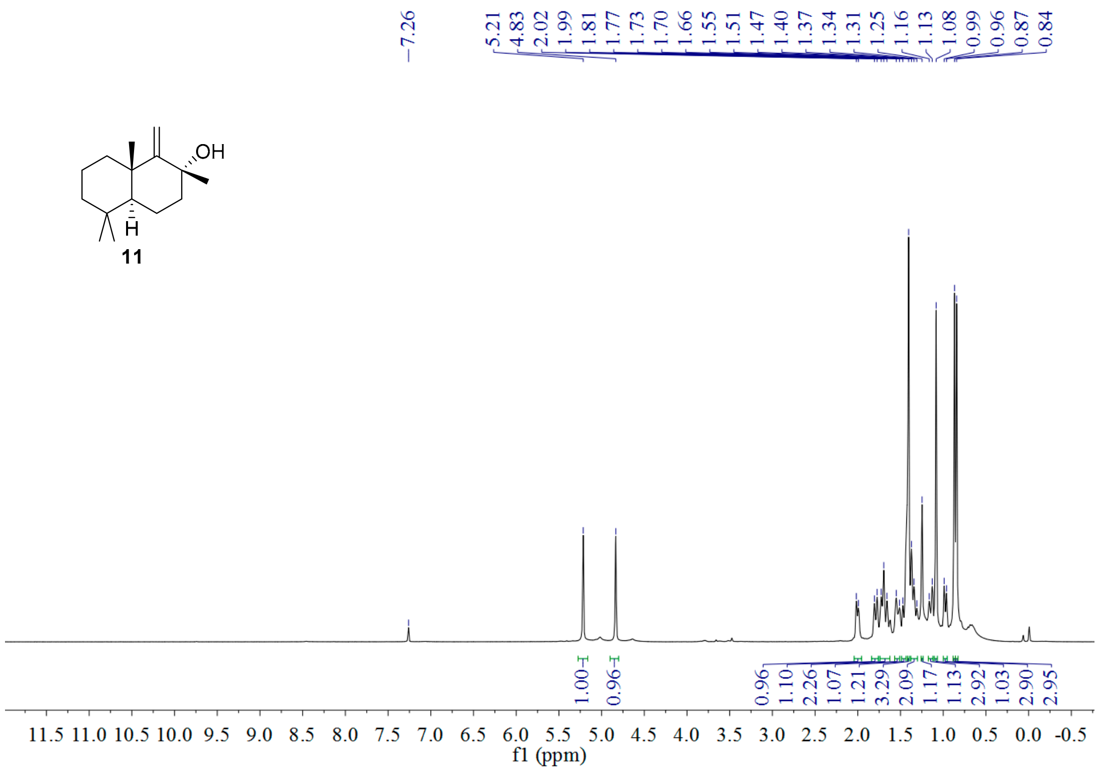

(**a**)

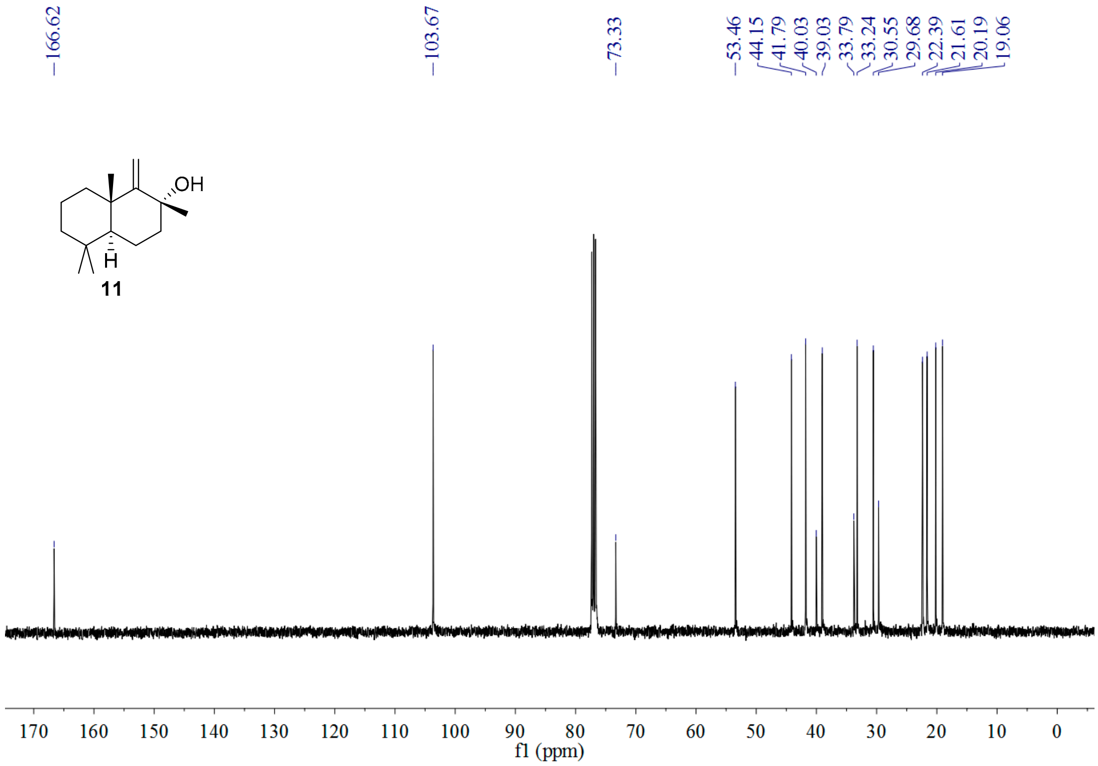

(**b**)

Figure S2. (**a**) ^{1}H NMR spectrum of compound **11**; (**b**) ^{13}C NMR spectrum of compound **11**.

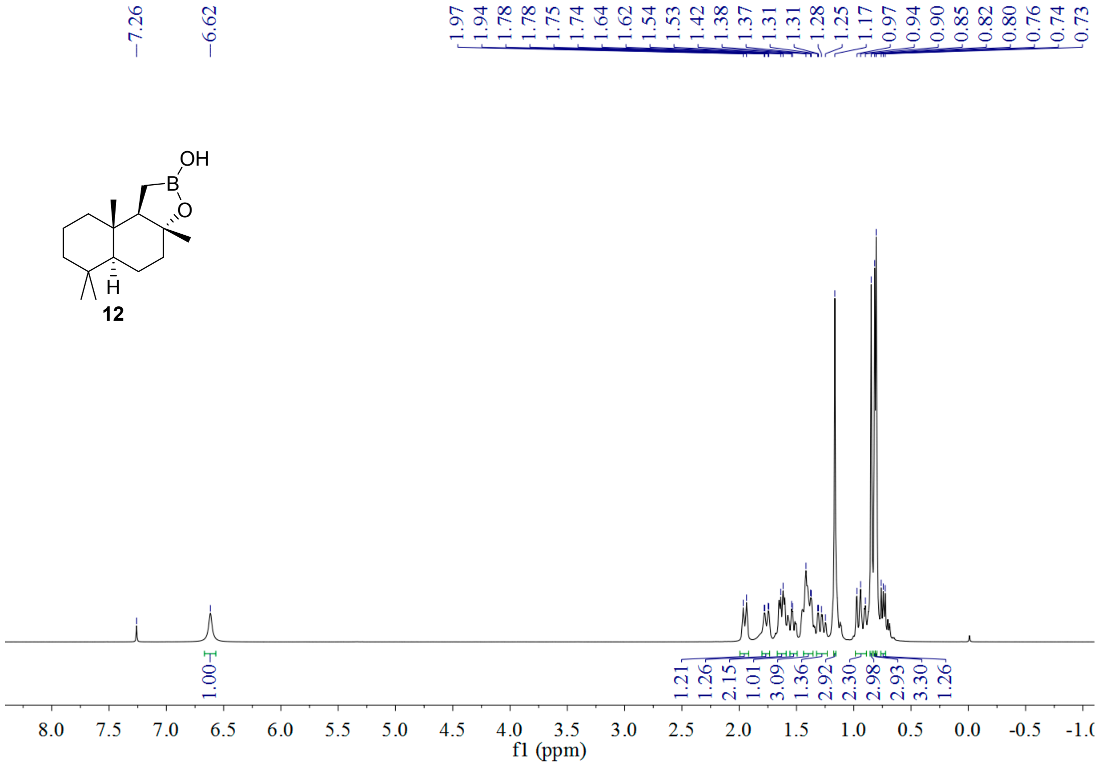

(**a**)

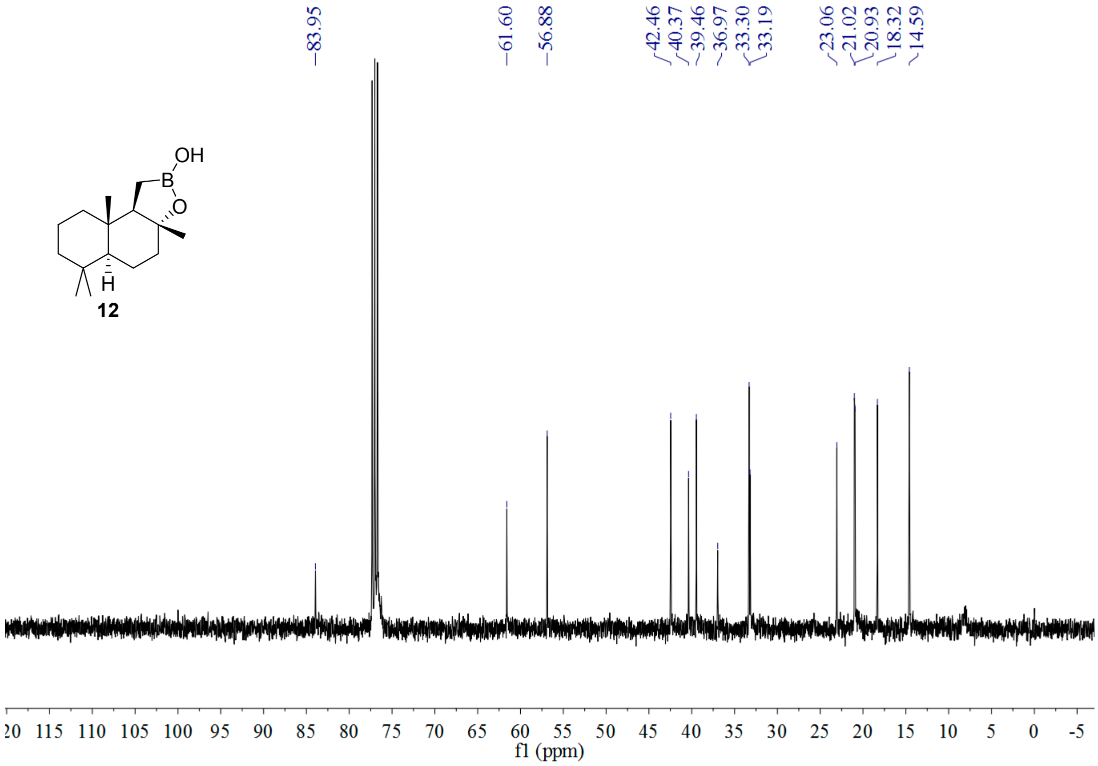

(**b**)

Figure S3. (**a**) ^{1}H NMR spectrum of compound **12**; (**b**) ^{13}C NMR spectrum of compound **12**.

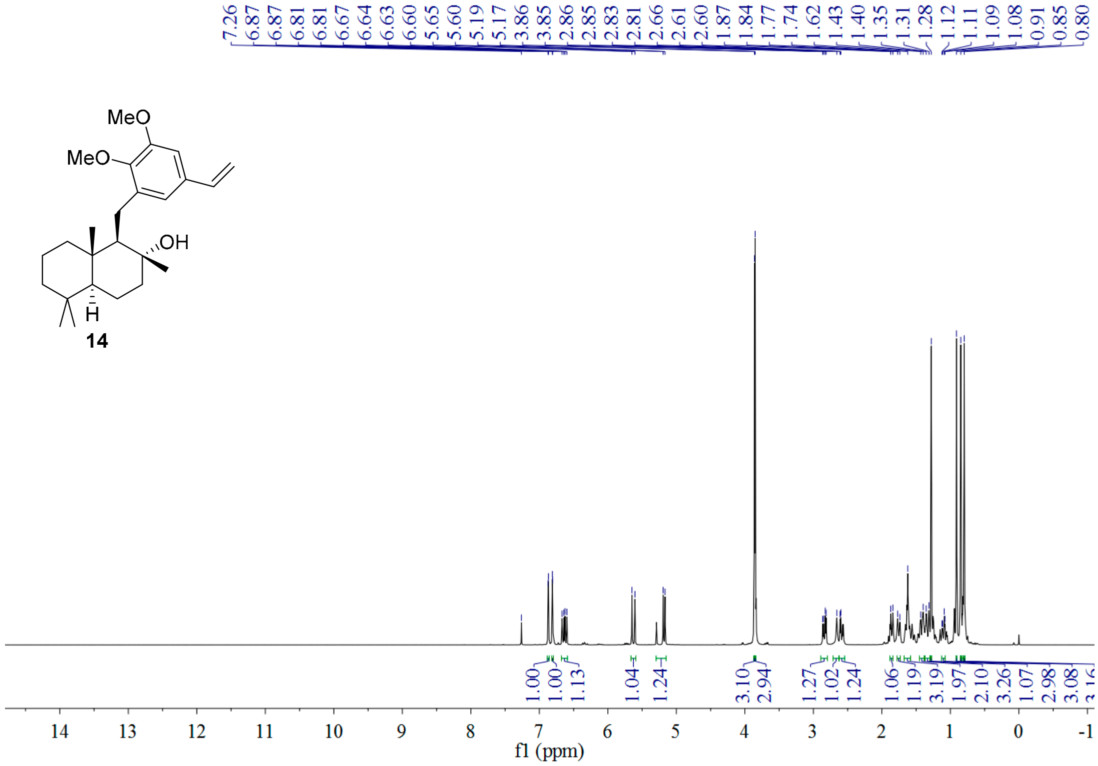

(**a**)

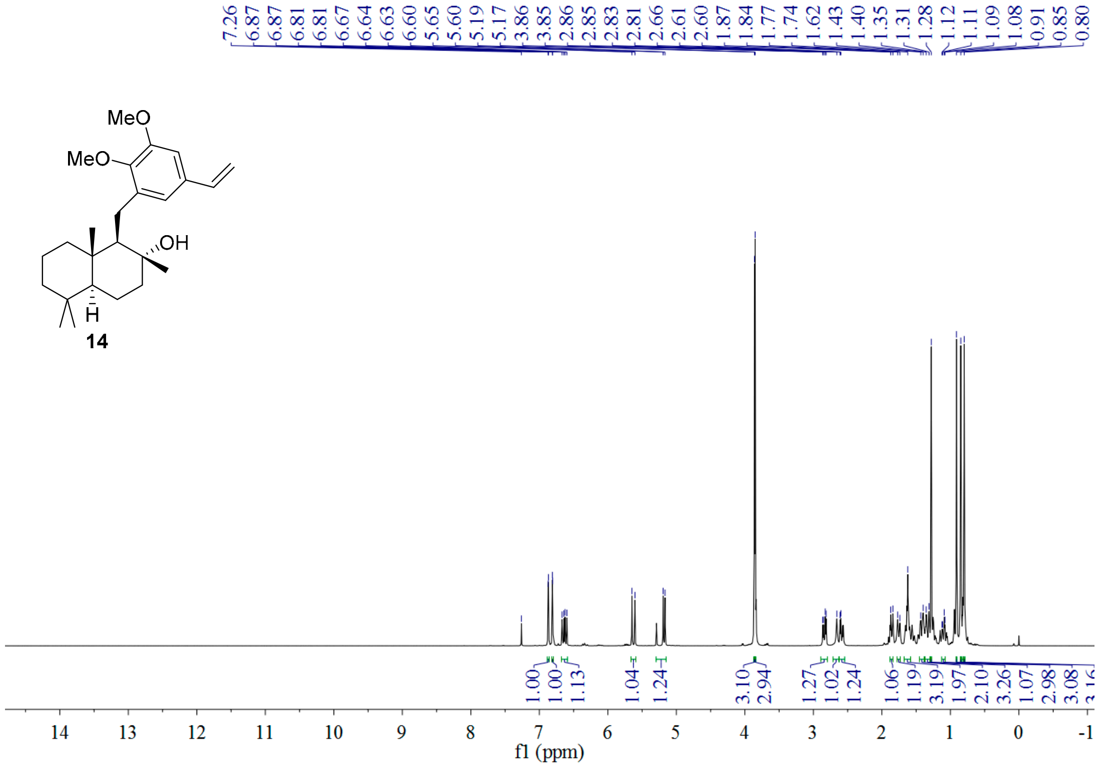

(**b**)

Figure S4. (**a**) ^{1}H NMR spectrum of compound **14**; (**b**) ^{13}C NMR spectrum of compound **14**.

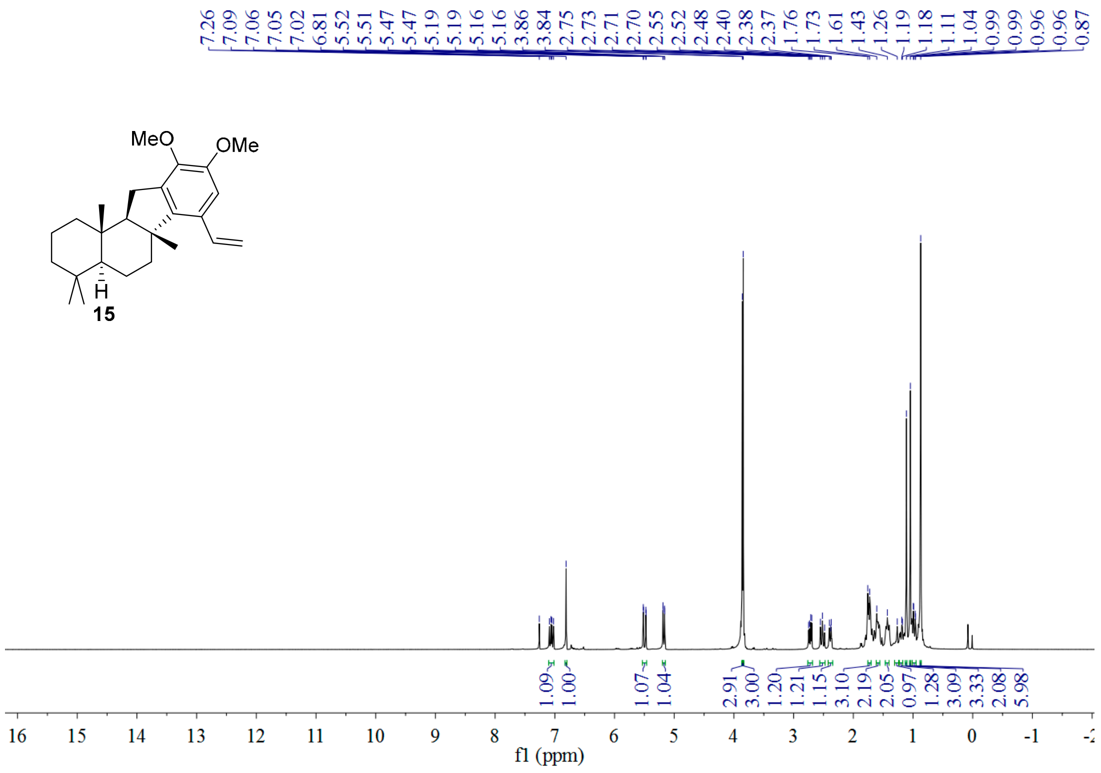

(**a**)

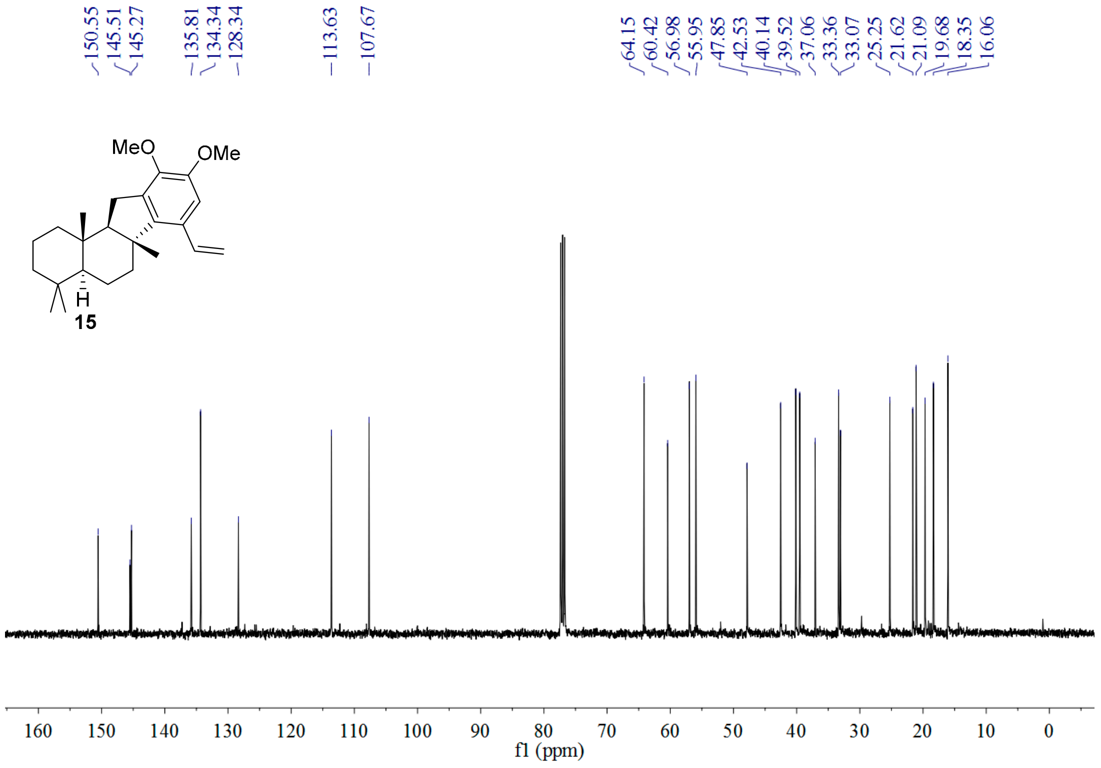

(**b**)

Figure S5. (**a**) ^{1}H NMR spectrum of compound **15**; (**b**) ^{13}C NMR spectrum of compound **15**.

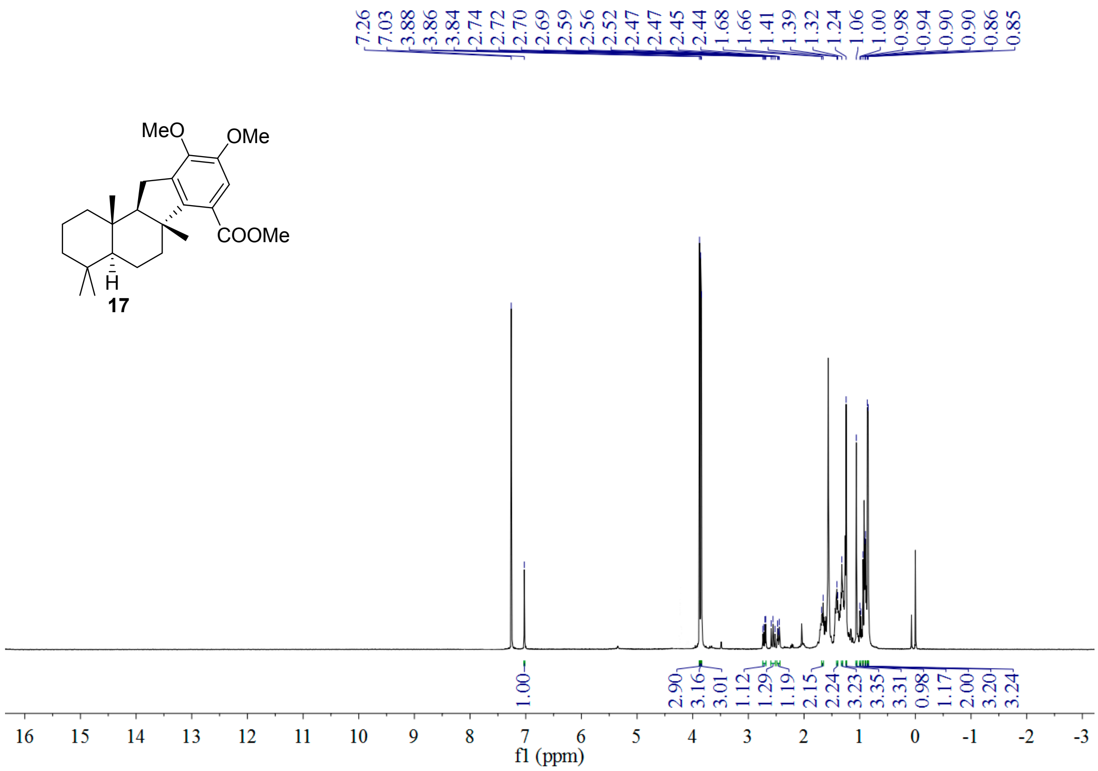

(**a**)

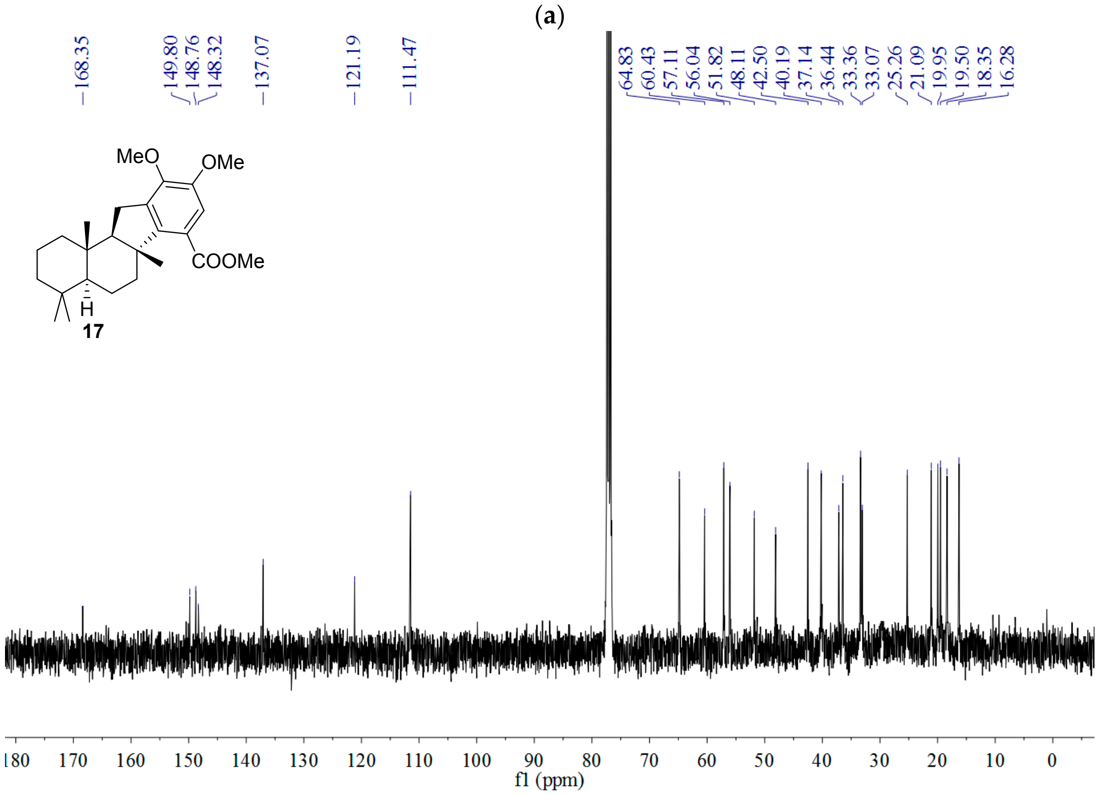

(**b**)

Figure S6. (**a**) ^{1}H NMR spectrum of compound **17**; (**b**) ^{13}C NMR spectrum of compound **17**.

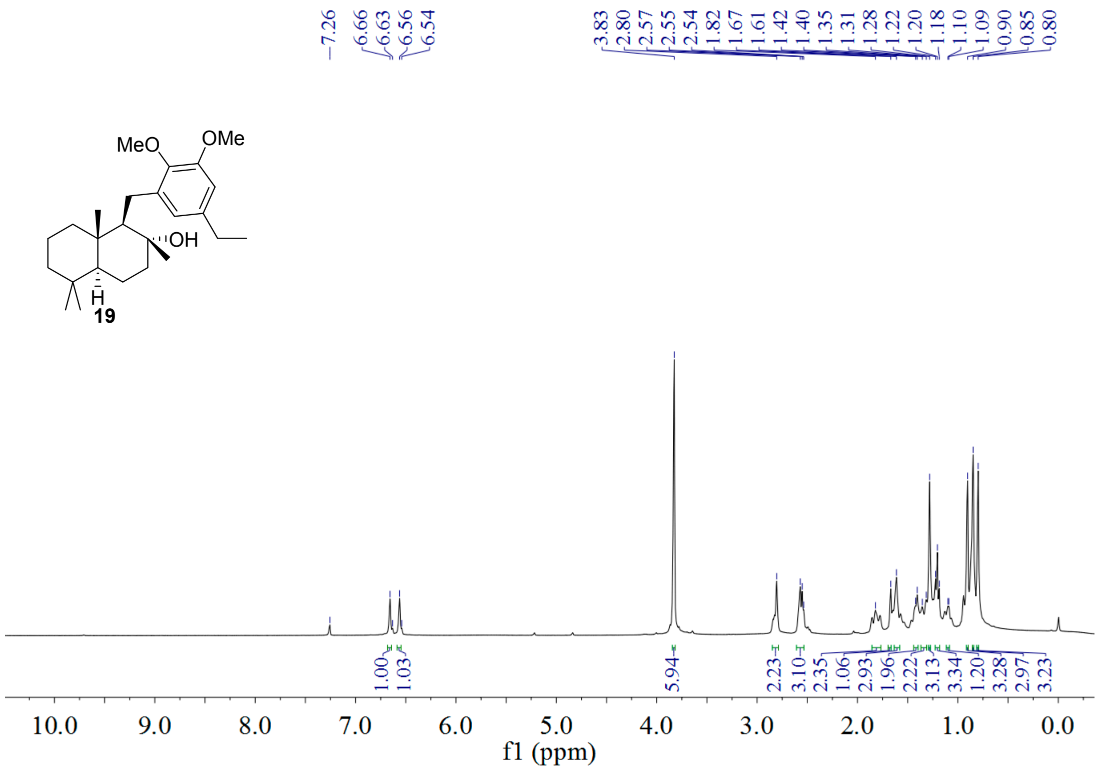

(**a**)

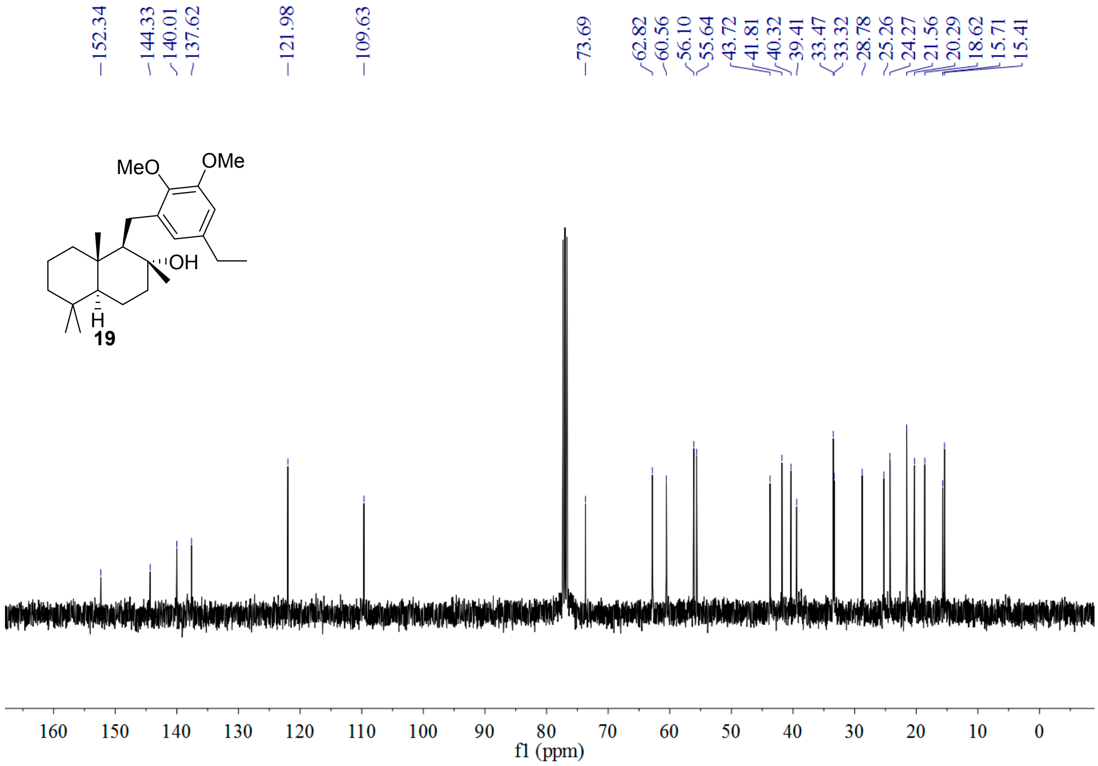

(**b**)

Figure S7. (**a**) ^{1}H NMR spectrum of compound **19**; (**b**) ^{13}C NMR spectrum of compound **19**.

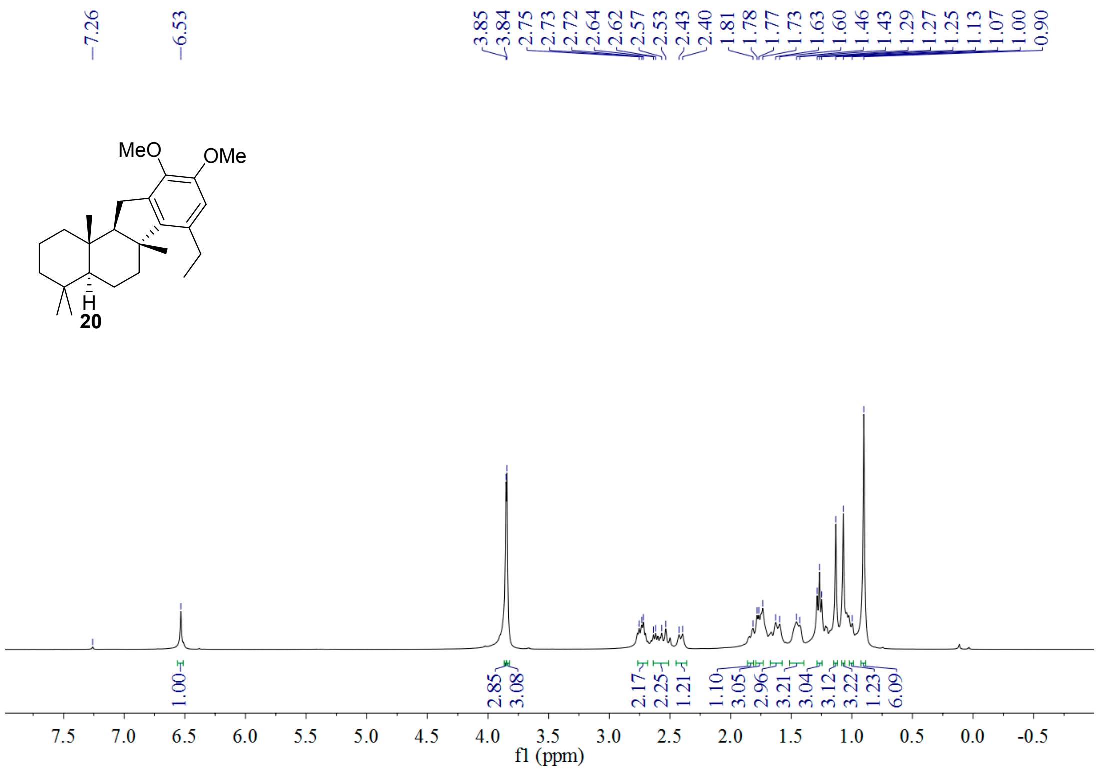

(**a**)

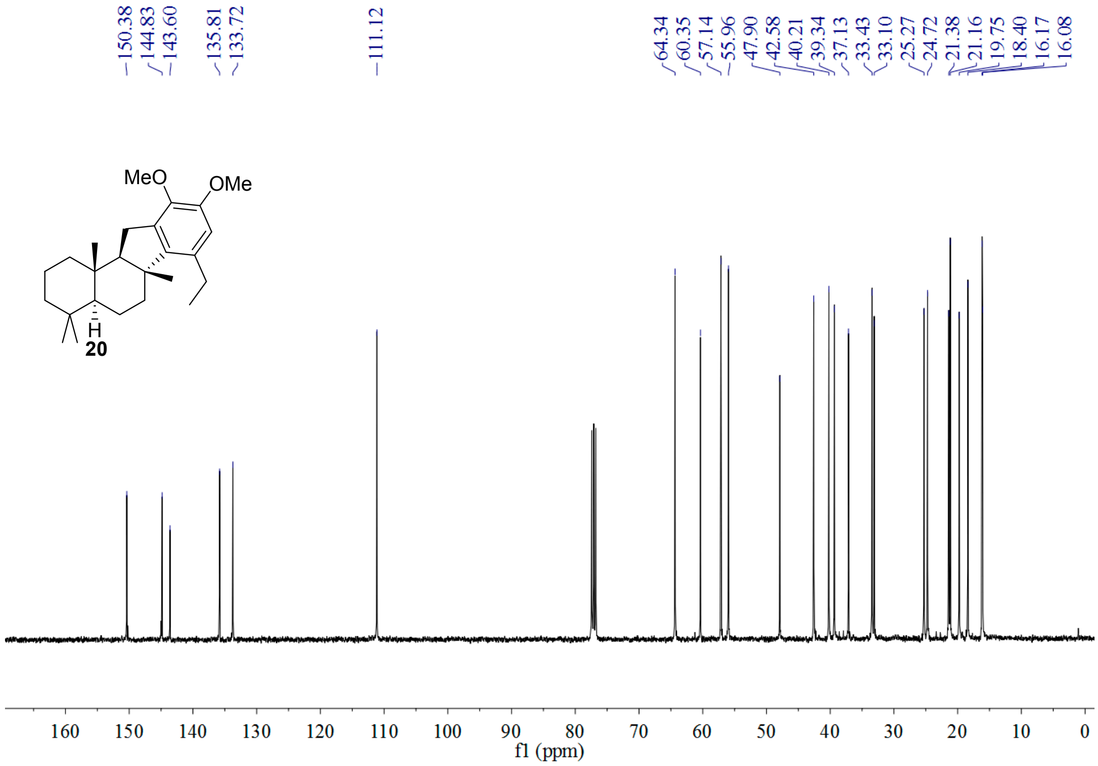

(**b**)

Figure S8. (**a**) ^{1}H NMR spectrum of compound **20**; (**b**) ^{13}C NMR spectrum of compound **20**.

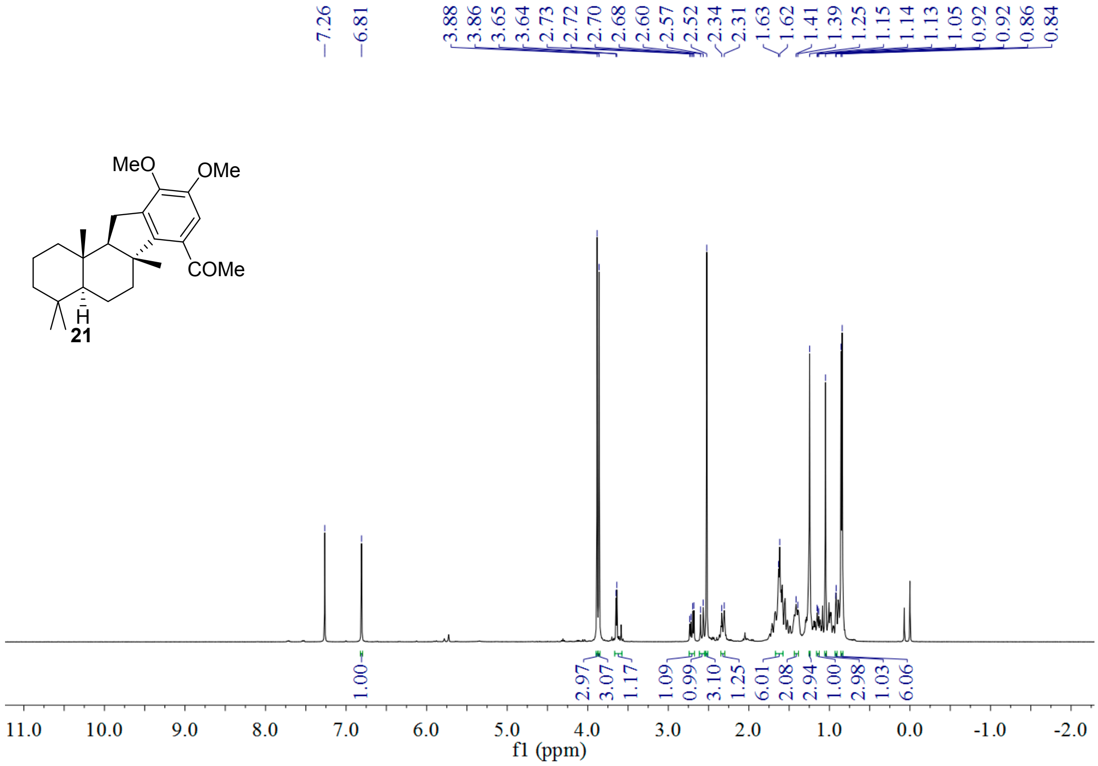

(**a**)

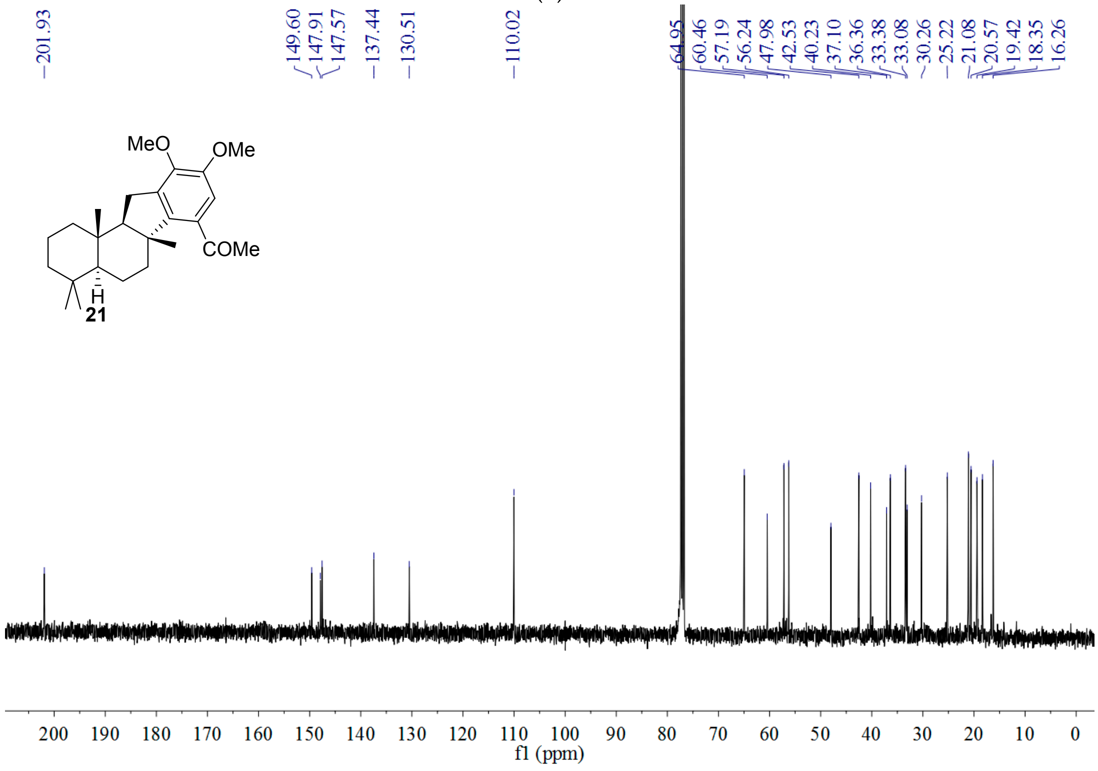

(**b**)

Figure S9. (**a**) ^{1}H NMR spectrum of compound **21**; (**b**) ^{13}C NMR spectrum of compound **21**.

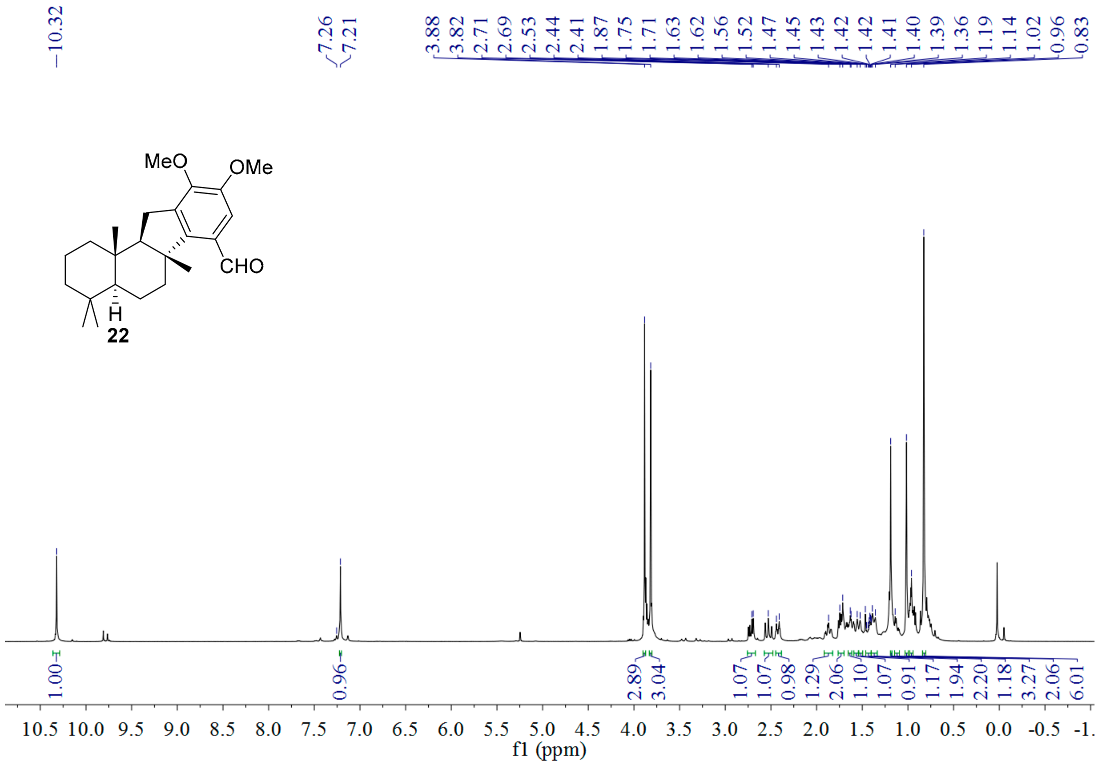

(**a**)

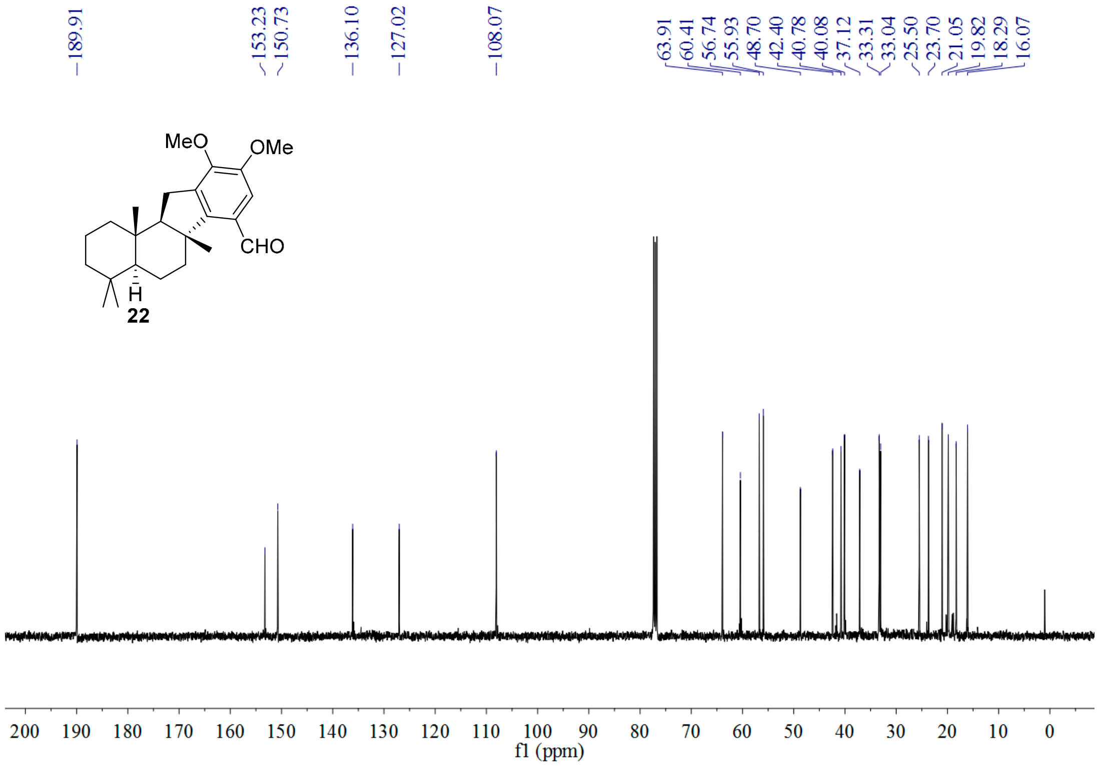

(**b**)

Figure S10. (**a**) ^{1}H NMR spectrum of compound **22**; (**b**) ^{13}C NMR spectrum of compound **22**.

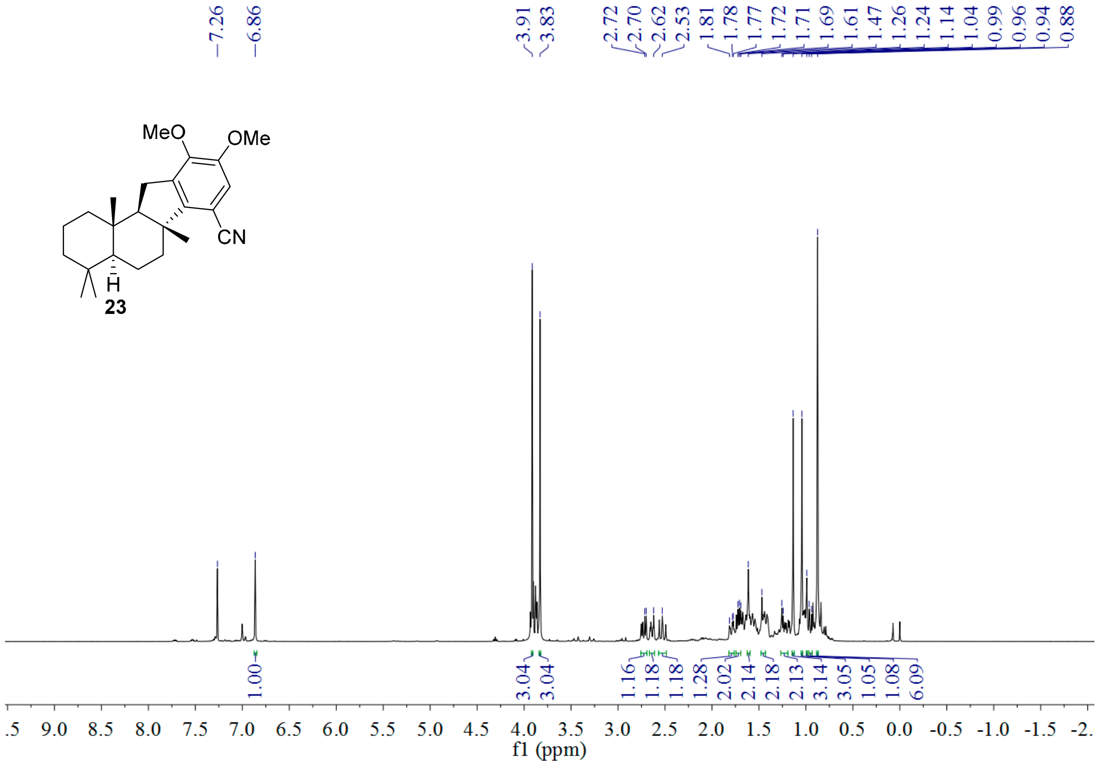

(**a**)

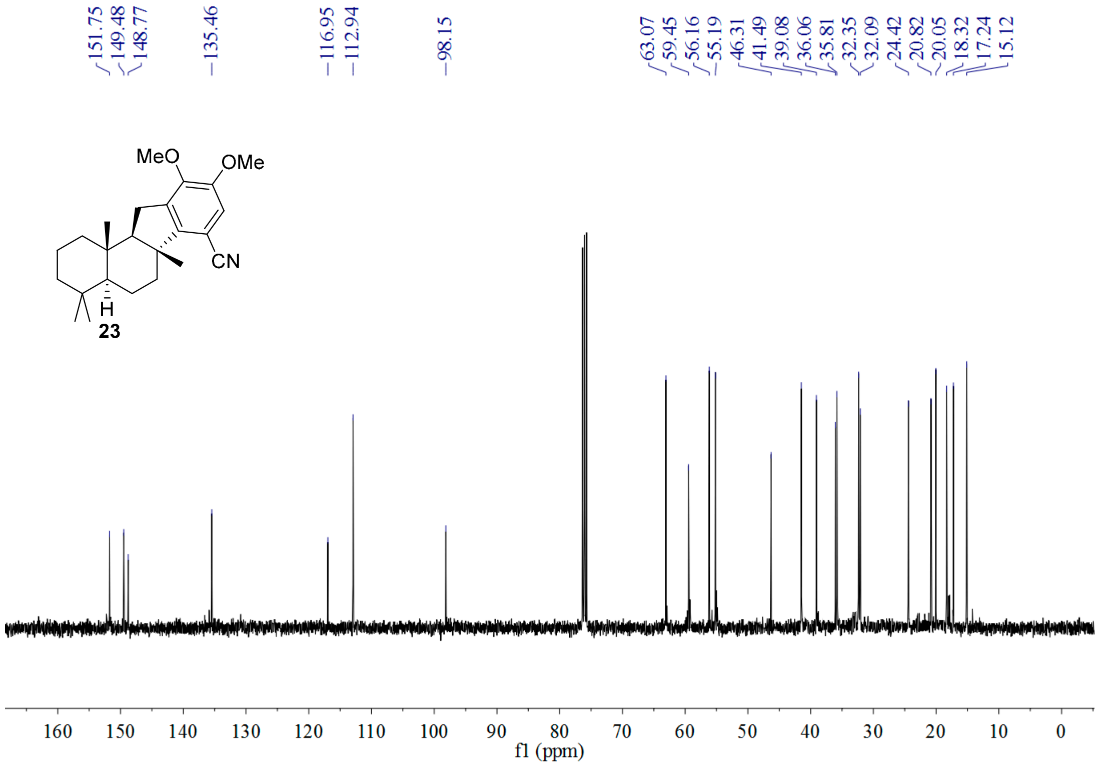

(**b**)

Figure S11. (**a**) ^{1}H NMR spectrum of compound **23**; (**b**) ^{13}C NMR spectrum of compound **23**.

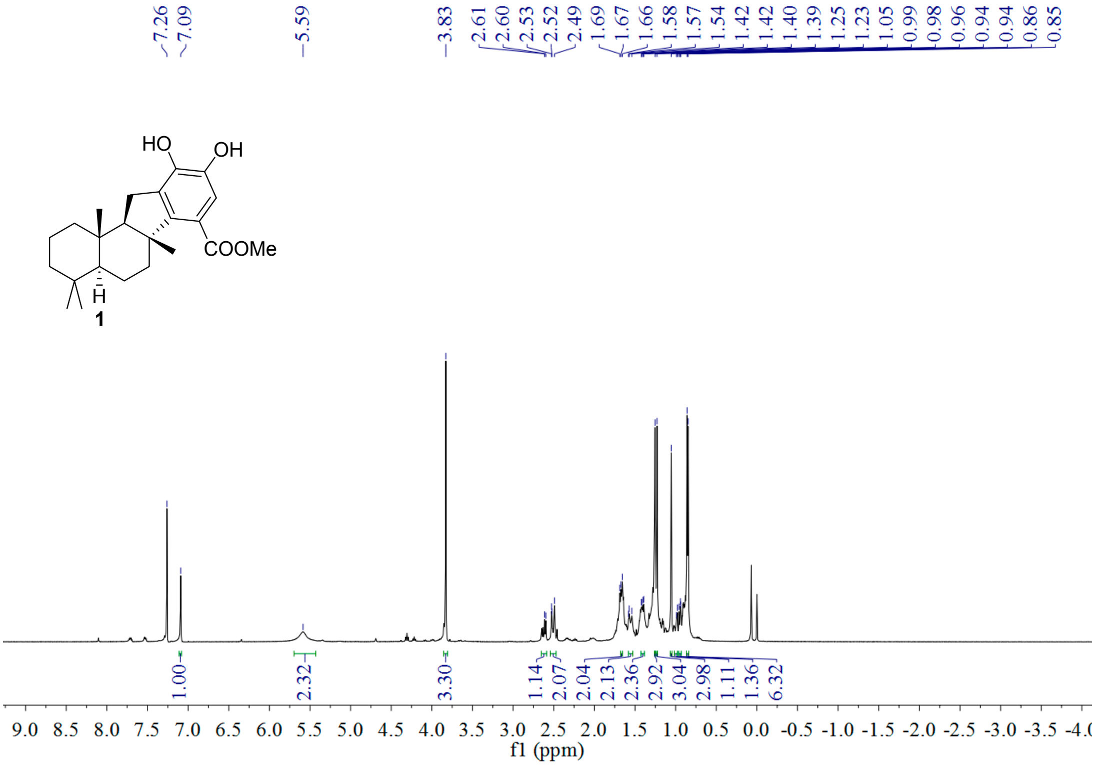

(**a**)

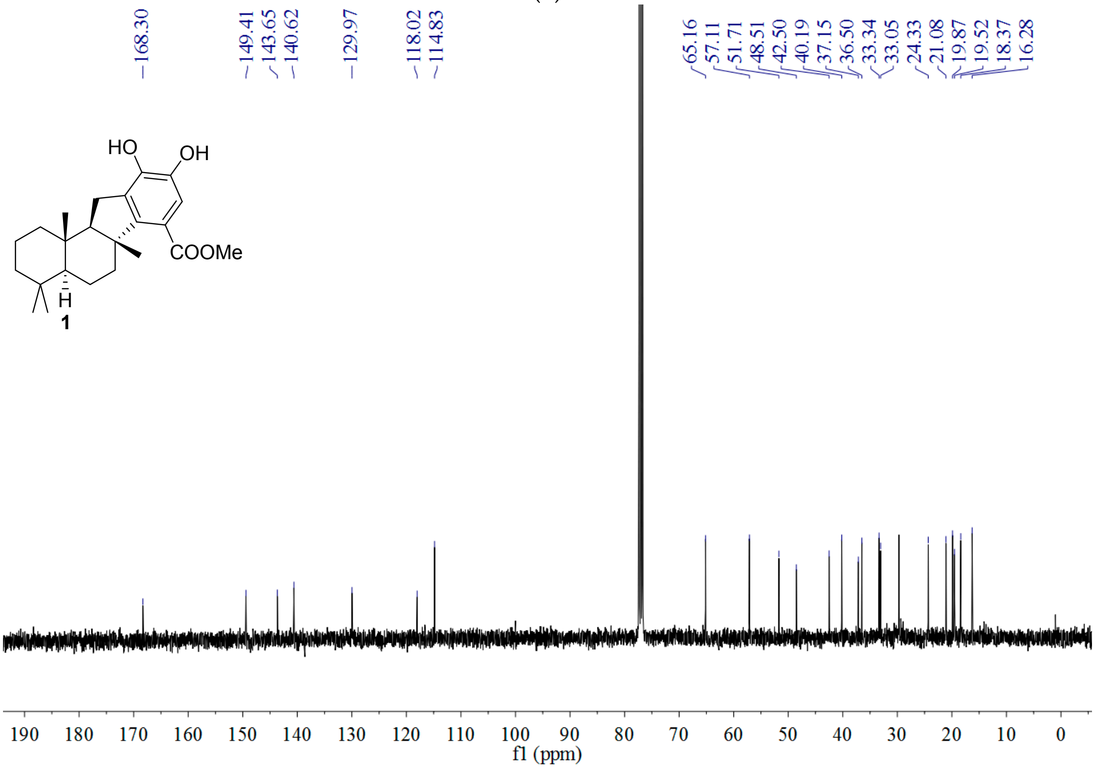

(**b**)

Figure S12. (**a**) ^{1}H NMR spectrum of compound **1**; (**b**) ^{13}C NMR spectrum of compound **1**.

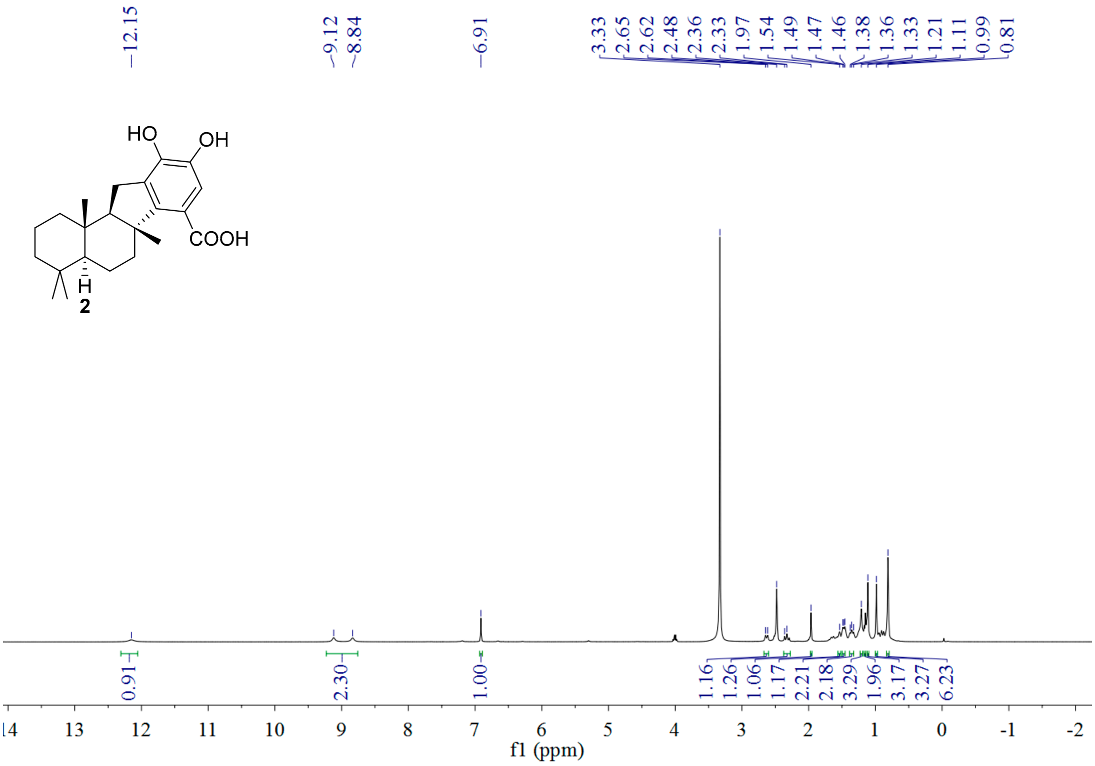

(**a**)

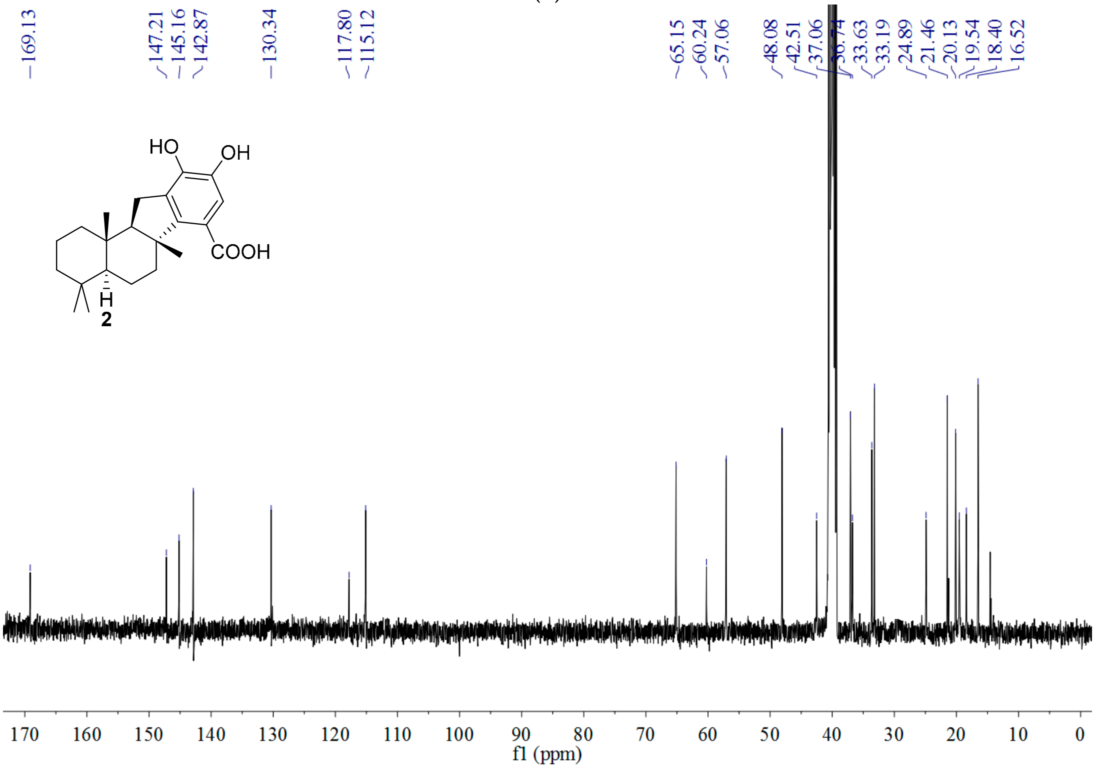

(**b**)

Figure S13. (**a**) ^{1}H NMR spectrum of compound **2**; (**b**) ^{13}C NMR spectrum of compound **2**.

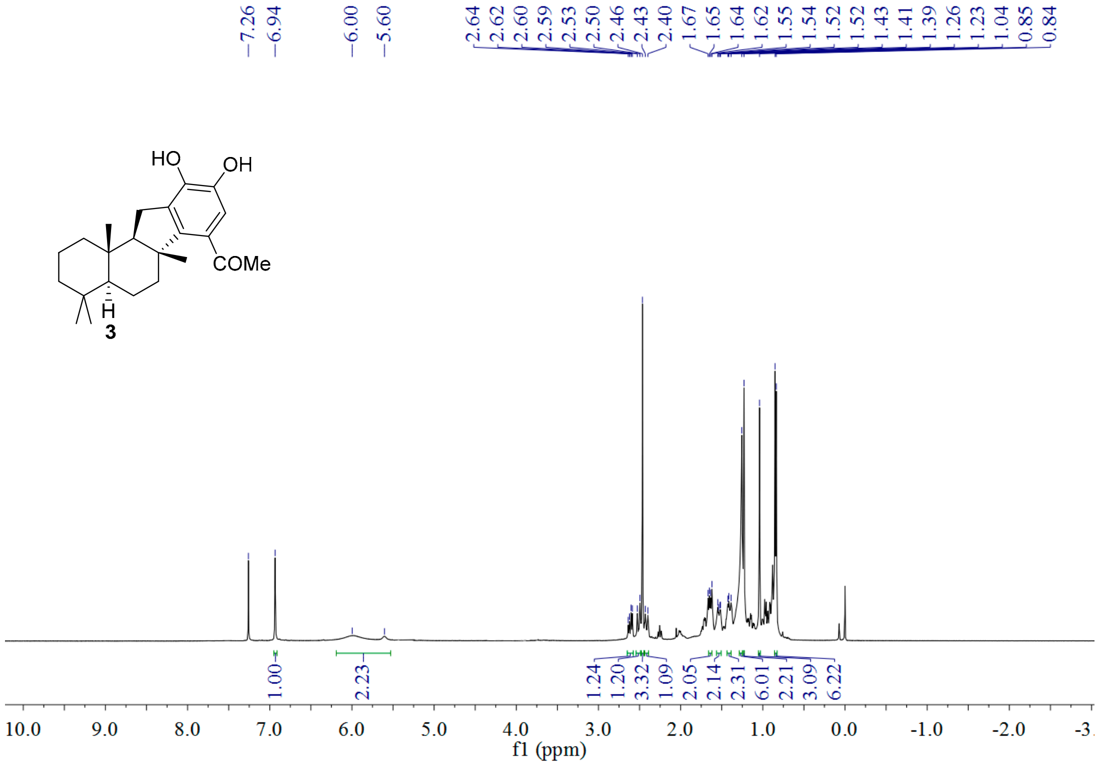

(**a**)

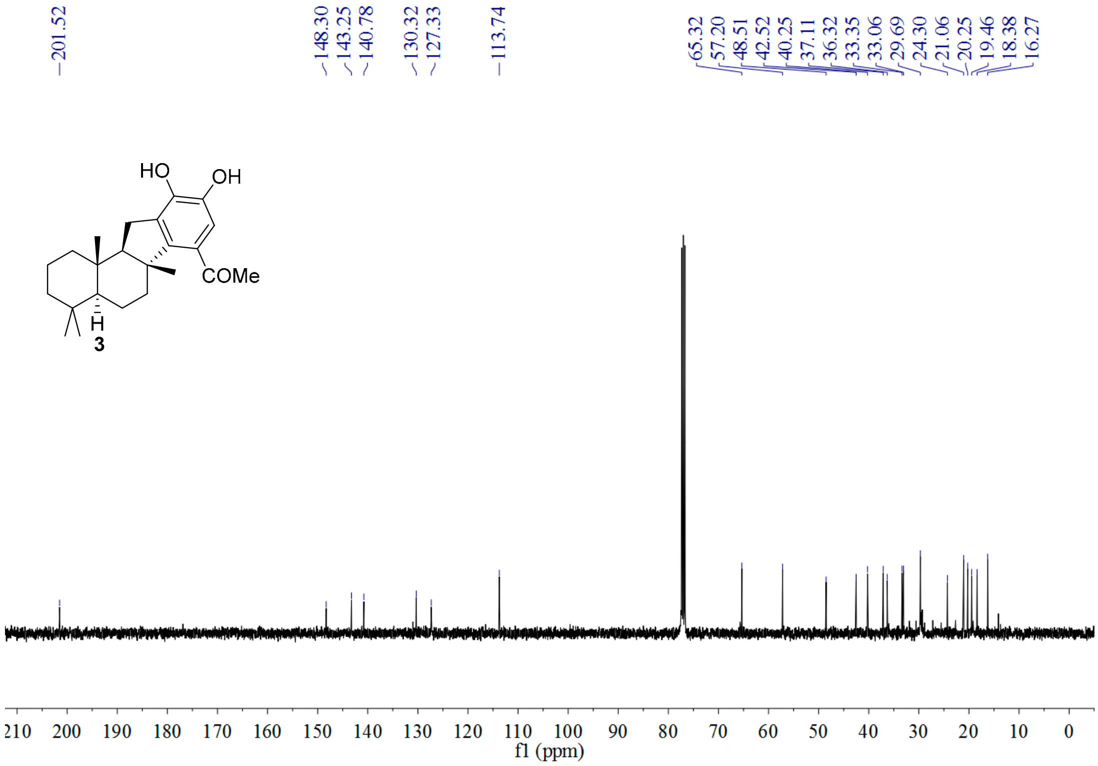

(**b**)

Figure S14. (**a**) ^{1}H NMR spectrum of compound **3**; (**b**) ^{13}C NMR spectrum of compound **3**.

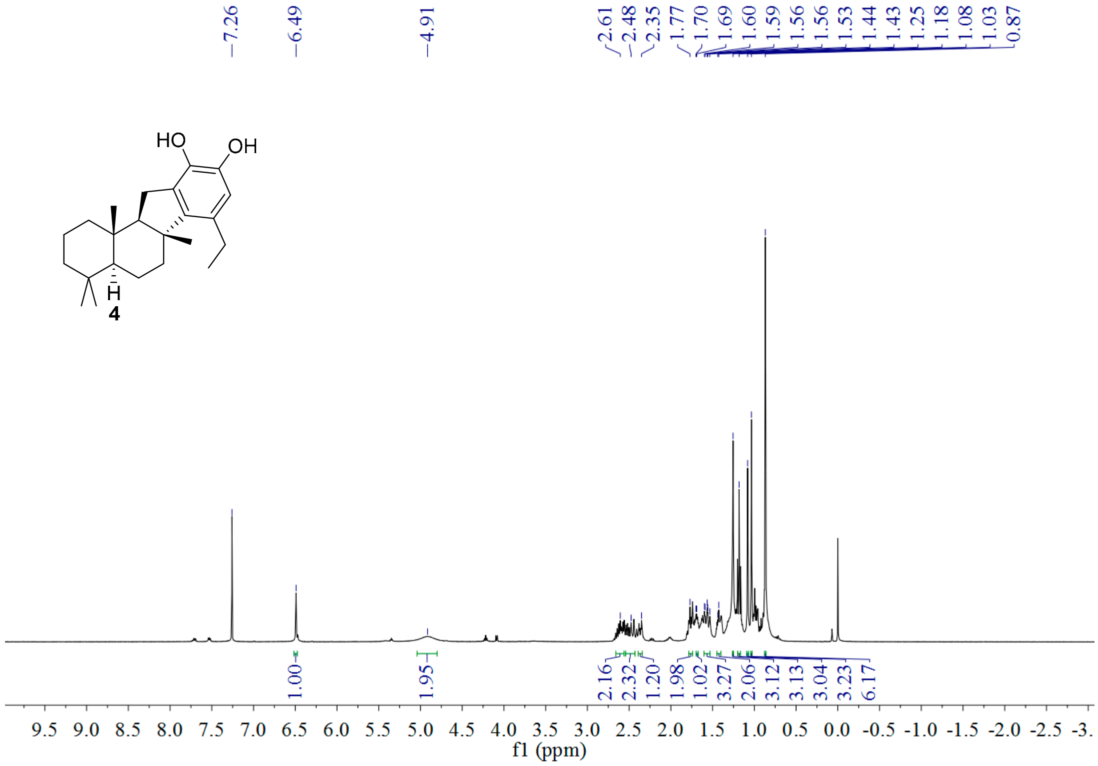

(**a**)

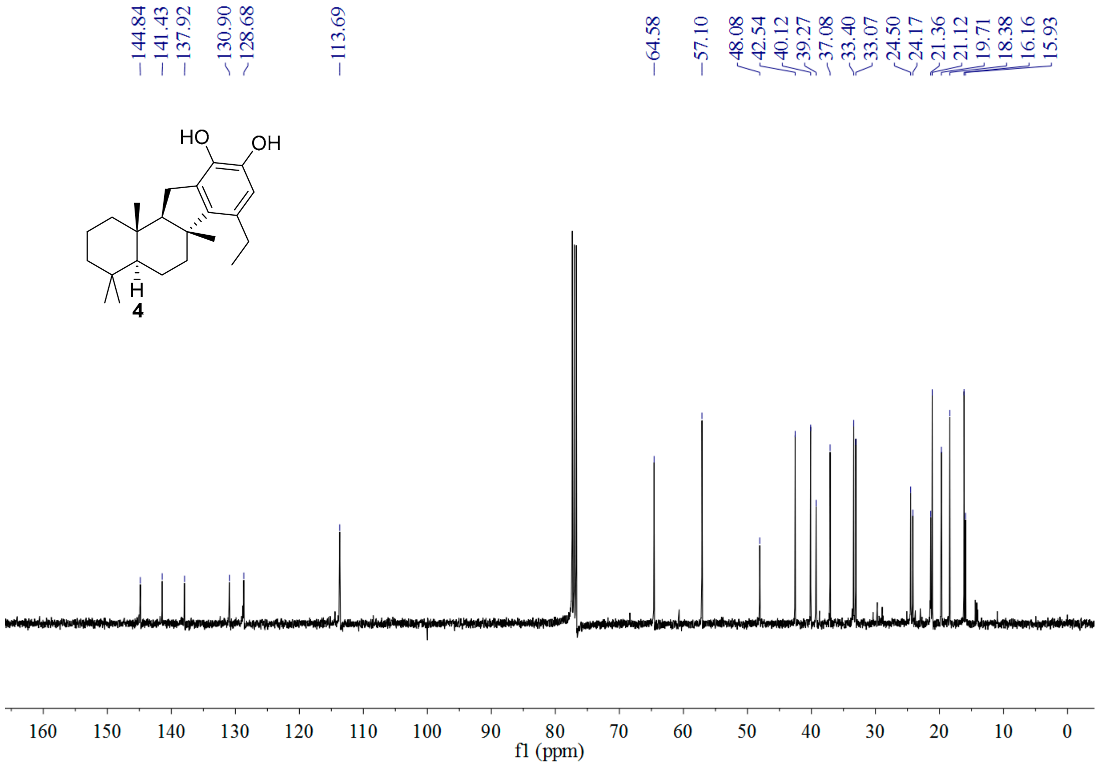

(**b**)

Figure S15. (**a**) ^{1}H NMR spectrum of compound **4**; (**b**) ^{13}C NMR spectrum of compound **4**.

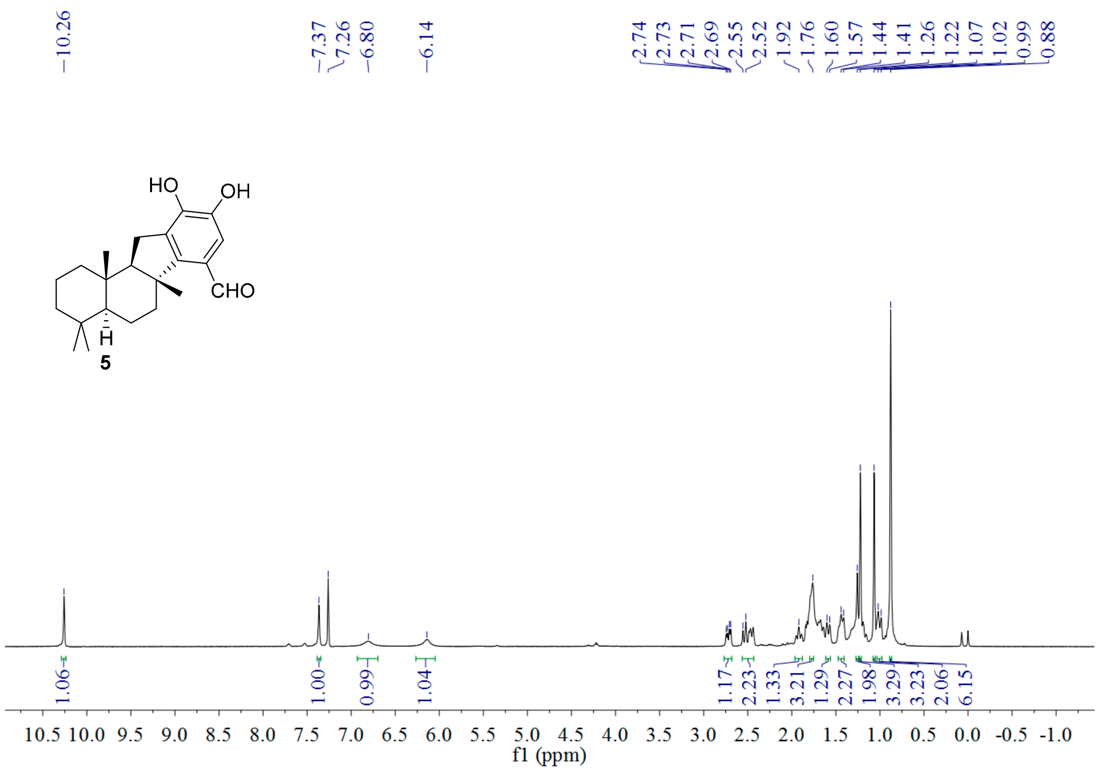

(**a**)

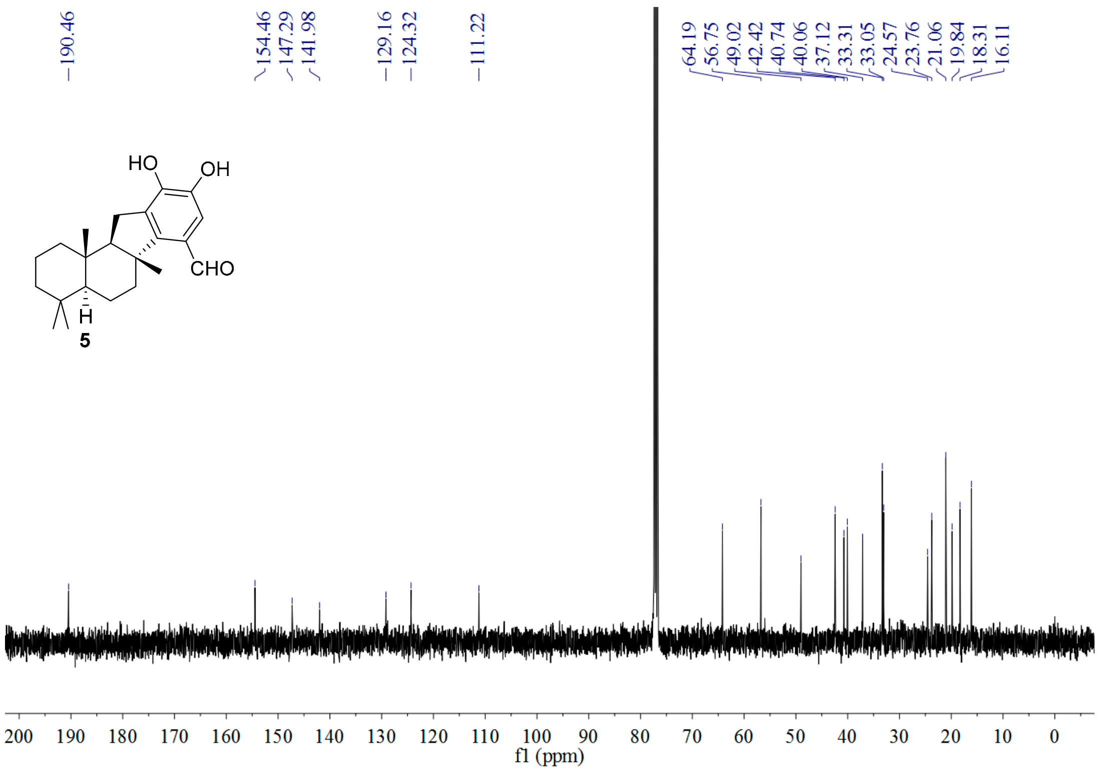

(**b**)

Figure S16. (**a**) ^{1}H NMR spectrum of compound **5**; (**b**) ^{13}C NMR spectrum of compound **5**.

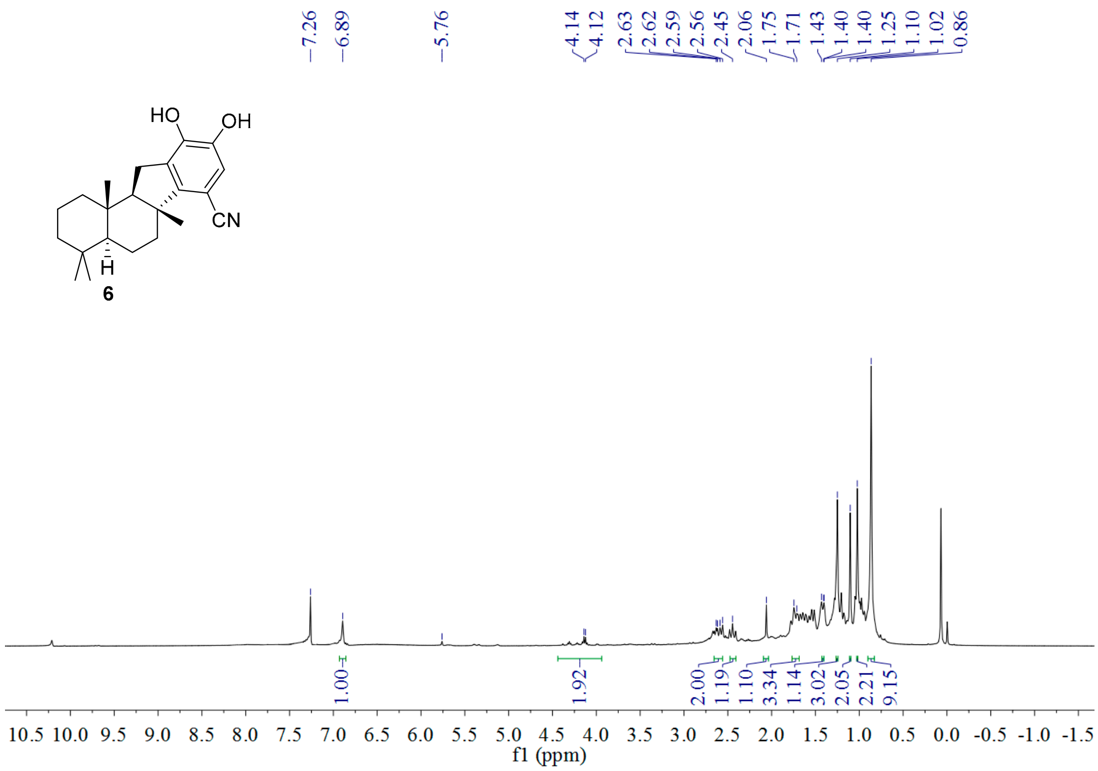

(**a**)

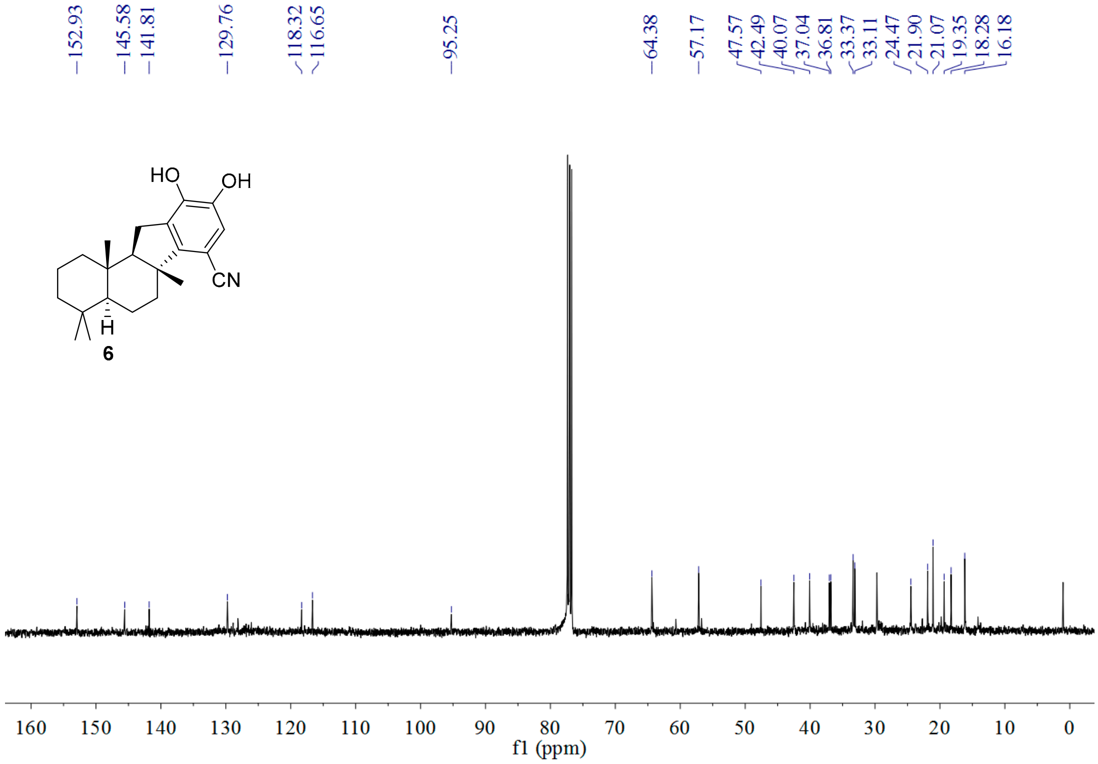

(**b**)

Figure S17. (**a**) ^{1}H NMR spectrum of compound **6**; (**b**) ^{13}C NMR spectrum of compound **6**.
